# Dealing with Varying Detection Probability, Unequal Sample Sizes and Clumped Distributions in Count Data

**DOI:** 10.1371/journal.pone.0040923

**Published:** 2012-07-20

**Authors:** D. Johan Kotze, Robert B. O’Hara, Susanna Lehvävirta

**Affiliations:** 1 Department of Environmental Sciences, University of Helsinki, Helsinki, Finland; 2 Department of Mathematics and Statistics, University of Helsinki, Helsinki, Finland; 3 Biodiversity and Climate Research Centre, Frankfurt am Main, Germany; 4 Botanic Garden, Finnish Museum of Natural History, University of Helsinki, Helsinki, Finland; Sapienza University of Rome, Italy

## Abstract

Temporal variation in the detectability of a species can bias estimates of relative abundance if not handled correctly. For example, when effort varies in space and/or time it becomes necessary to take variation in detectability into account when data are analyzed. We demonstrate the importance of incorporating seasonality into the analysis of data with unequal sample sizes due to lost traps at a particular density of a species. A case study of count data was simulated using a spring-active carabid beetle. Traps were ‘lost’ randomly during high beetle activity in high abundance sites and during low beetle activity in low abundance sites. Five different models were fitted to datasets with different levels of loss. If sample sizes were unequal and a seasonality variable was not included in models that assumed the number of individuals was log-normally distributed, the models severely under- or overestimated the true effect size. Results did not improve when seasonality and number of trapping days were included in these models as offset terms, but only performed well when the response variable was specified as following a negative binomial distribution. Finally, if seasonal variation of a species is unknown, which is often the case, seasonality can be added as a free factor, resulting in well-performing negative binomial models. Based on these results we recommend (a) add sampling effort (number of trapping days in our example) to the models as an offset term, (b) if precise information is available on seasonal variation in detectability of a study object, add seasonality to the models as an offset term; (c) if information on seasonal variation in detectability is inadequate, add seasonality as a free factor; and (d) specify the response variable of count data as following a negative binomial or over-dispersed Poisson distribution.

## Introduction

A major aspect of measuring biodiversity is simply estimating the abundance of different species, whether as the actual number in an area or measuring relative abundance, so that different areas can be compared. Many biodiversity surveys use the latter approach by trapping or observing individuals in an area. These surveys and monitoring programs must incorporate two major sources of variation when sampling biological organisms: spatial variation and detectability [1]. This paper deals with the latter. Simple analyses will assume that detectability is the same, but for most organisms, detectability varies over time due to variation in seasonal or diurnal activity [2]–[8]. For example, bark-foraging birds prefer to forage in woodland interior habitat and on large diameter trees during the breeding season, but not during the non-breeding season [9], small orb-weaving spiders build their webs early in the evening while larger spiders put up their webs throughout the night [10], and many European carabid beetles are active in either the spring or autumn, but others are active throughout the snow-free period [11]–[12]. Detectability can even vary for plants, which may remain below the soil surface for part of their annual cycle with most biomass in the roots, or may be present only as small rosettes outside the flowering period.

Irrespective of the kind of abundance (true, relative, activity-density or other indices) reflected by the data (see [13]), seasonal variation in detectability can cause biases in the analysis of data and the subsequent interpretation, so either the collection of data or its analysis needs to control for it [14]. One way of avoiding this problem is to only sample organisms when the probability of observing individuals is constant. For example, the cover of an early spring flower can be measured only in early spring. Or, if the behavior and detectability of a species is influenced by the weather, observations could be made only during certain weather conditions (e.g., only collecting butterflies on sunny days). When conducting field studies, however, it is not always possible to control for this variation in detectability. Often sampling periods need to be long enough to collect a sufficient number of individuals in order to make meaningful inferences about populations. If this is the case, variation in the probability of observing individuals has to be controlled for at the data processing level, i.e. statistically. Methods exist to estimate detectability in the field, e.g. distance sampling and mark-recapture studies [13], [15], [16], but these are not appropriate for many organisms, and often it is enough to estimate relative abundance (e.g. to compare different habitat types), as it is not possible to carry out the extra work needed to estimate detectability directly.

Another problem related to the collection of ecological field data is that sample sizes may vary from one time or place to the next. Even with the best-prepared field experiments, ecologists are often faced with unbalanced designs. Designs may be unavoidably unbalanced from the start, for example because an investigator cannot make simultaneous observations at multiple localities and is consequently forced to sample different sites at different times. Samples may also become lost during the observation period, e.g. traps may be lost or broken or observers are unable to carry out all the observations required. If the experimental design is unbalanced, and, equally importantly, if the study organism varies in detectability over time (e.g., seasonal variation in activity), sampling effort may not be comparable between treatments. Simply stated, if samples are lost at different times during the field period (a common feature of studies in urban environments for example, see also [17]), pooling and standardizing the remaining samples over the whole field period may produce gross over- and underestimates of abundance and its variation, at least for species that are abundant or easily detectable only during some part of the season.

**Table 1:** Parameters used in data simulations.

Name	Mean*	Site Variance	Trap Variance	Results
Field Data	1.47	0.14^2^	0.30^2^	Figs. 1, 2
Low Mean	1.47/5	0.14^2^	0.30^2^	Fig. 3 & Supporting Information S2
High Mean	5×1.47	0.14^2^	0.30^2^	Fig. 3 & Supporting Information S2
Low Site, Low Trap Variances	1.47	0.14^2^/5	0.30^2^/5	Fig. 3 & Supporting Information S2
Low Site, High Trap Variances	1.47	0.14^2^/5	5×0.30^2^	Fig. 3 & Supporting Information S2
High Site, Low Trap Variances	1.47	5×0.14^2^	0.30^2^/5	Fig. 3 & Supporting Information S2
High Site, High Trap Variances	1.47	5×0.14^2^	5×0.30^2^	Fig. 3 & Supporting Information S2

‘Field Data’ parameters of *P. oblongopunctatus* are from [26]. By varying the mean estimated catch and variance at the treatment site and trap levels, six additional conditions were created for evaluation. The last column lists the locations of the results.

* means of Treatment 1.

**Table 2:** Created carabid beetle abundance datasets.

	Treatment level 1	Treatment level 2	Treatment level 3
Time Interval 1	7.4	**14.7**	**29.4**
Time Interval 2	14.4	**28.8**	**57.6**
Time Interval 3	3.8	7.6	15.3
Time Interval 4	**2.6**	**5.3**	10.6
Time Interval 5	**1.2**	**2.4**	4.7
Total number of individuals	29.4 (average catch: 1.47individuals/trap)	58.8 (average catch: 2.94individuals/trap)	117.6 (average catch: 5.88individuals/trap)

Values present the average total catch per treatment per time interval. The datasets were created using seasonal data collected on *P. oblongopunctatus* (see text). Each treatment consisted of five replicates, with four traps per replicate (60 traps in total). The traps were visited five times (20 days intervals), resulting in 300 trapping events in the dataset. The whole procedure was repeated 100 times to create 100 carabid abundance datasets. Treatment levels 2 and 3, Time Intervals 1 and 2 (“High catch loss”) and Treatment levels 1 and 2, Time Intervals 4 and 5 (“Low catch loss”) represent the cells from where traps were randomly lost (see text).

In this paper we evaluate the effects of varying detectability and sampling effort on the statistical analyses of count data. We also show the merits of modeling the response variable as following a negative binomial distribution [18], compared to often-used Gaussian equivalents. Briefly, ecological field data often consist of counts (i.e. discrete, such as number of individuals or species in a patch, number of offspring, or number of parasites per host), which are typically heteroscedastic (i.e. the variance varies across samples). Rather than transforming the data, which may not always work [19] – as with many zeroes the residuals must be skewed [20] and thus the assumption of normality is suspect – a preferable strategy would be to use models developed for count data. A Poisson distribution will be a reasonable starting assumption, but for most ecological data (see [8]) the clumped, or aggregated nature of the measurement variable (e.g. individuals of a species), inflates the sample variance over what a Poisson distribution would assume [21]. There are several approaches to overcoming this; adding an over-dispersion term [22], using a quasi-likelihood model [22], [23], or using another distribution that incorporates extra variation, e.g. the negative binomial distribution [18], [19].

## Materials and Methods

We based our simulation on the activity of *Pterostichus oblongopunctatus* F., a ground beetle species (Carabidae) that occurs in our study sites in Finland, but stress that the methods discussed can be applied to a wide range of ecological data. These beetles are often collected using pitfall traps, and sampling is usually continuous from early May to the end of September. *P. oblongopunctatus* is a spring-active beetle, so its abundance and resultant detectability decreases during the trapping period. Previously collected field data [24]–[29] show that approximately 25% of the individuals of *P. oblongopunctatus* are collected during the first 20 days of sampling (trapping of beetles usually starts in the first or second week of May), followed by 49%, 13%, 9% and 4% during the subsequent 20-day periods over a 100-day continuous sampling period: we interpret this variation in proportions caught as variation in activity.

**Figure 1:**
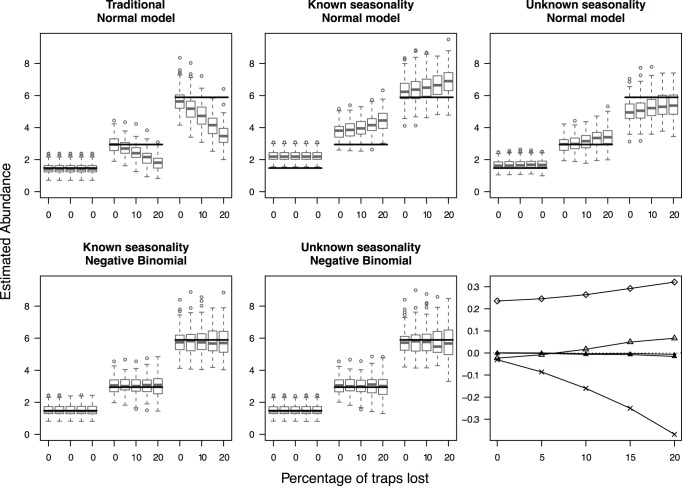
Predicted catch after trap losses at high activity in high-abundance treatments. Box and whisker plots of the effect sizes (predicted catch) of the analyses performed with five models for original parameters estimated from data on *Pterostichus oblongopunctatus* abundances, and trap loss at high activity in treatments 2 and 3. The black horizontal lines represent the simulated (i.e. true) total abundances per treatment (3 treatments) without trap loss. The x-axis represents the three Treatment levels with five states of trap loss per treatment (from no loss to 20% loss across the whole design). Since no trap losses occurred at the low-abundance treatment (treatment 1), losses were zero for the first five box and whisker plots. The last panel represents the mean bias of the models against trap loss (see Fig. 3).

**Figure 2:**
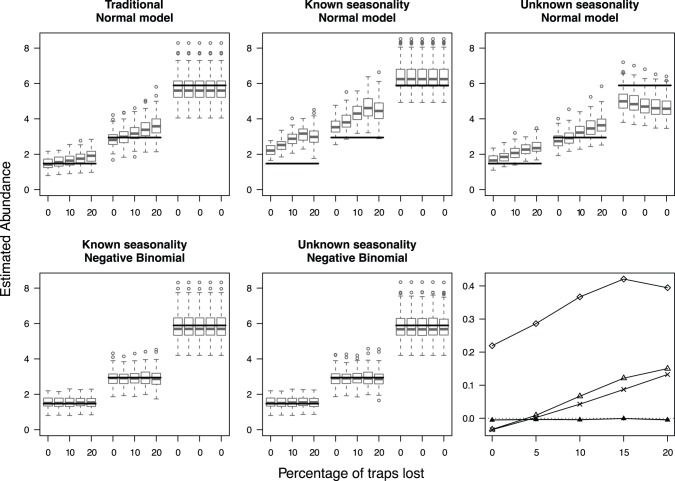
Predicted catch after trap losses at low activity in low-abundance treatments. Box and whisker plots of the effect sizes (predicted catch) of the analyses performed with five models for original parameters estimated from data on *Pterostichus oblongopunctatus* abundances, and trap loss at low activity in treatments 1 and 2. Since no trap losses occurred at the high-abundance treatment (treatment 3), losses were zero for the last five box and whisker plots. See Figs. 1 and 3 for more details.

We fitted a model to field data from catches of *P. oblongopunctatus* [26]: time period, replicate plot and trap were used as random effects, with the response assumed to be quasi-Poisson with a log link function. This is equivalent to assuming that the random effects were log-normally distributed. The estimated standard deviations were 0.14 and 0.30 for site and trap respectively, and the mean catch was set to the average mean over the whole season (see first row in Table 1).

**Figure 3:**
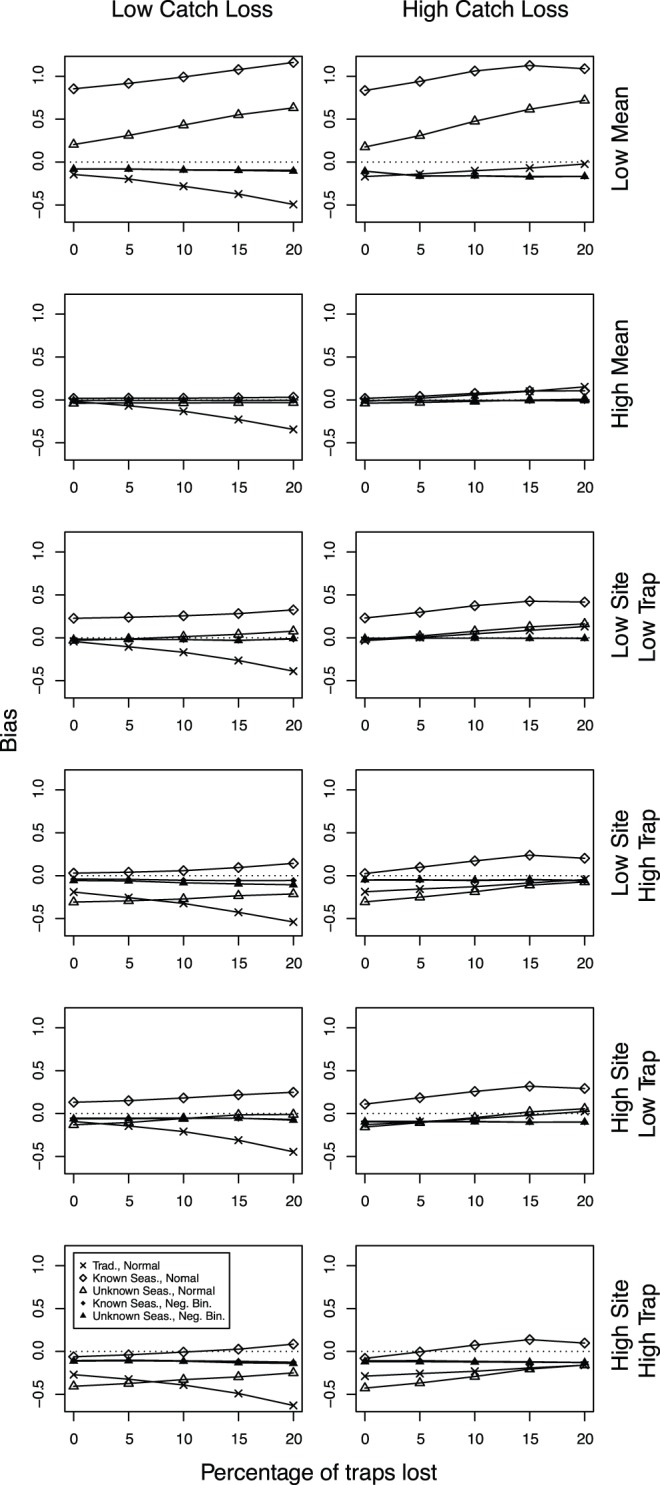
Mean bias of the models. Plots of mean bias of the models against trap loss for models with different mean abundances and variances in abundance (see rows 2–7 in Table 1).

From this set of parameters, we created several sets of simulated data. For each we simulated three treatments, each measured in five replicate plots, each with four pitfall traps. The second and third treatments had means two and four times that of the first treatment respectively (see last row in Table 2). The cumulative expected catch over the sampling period was divided between five time intervals as specified by the percentages above (i.e. 25, 49, 13, 9, 4) (columns in Table 2). The expected catch was then equally split between the five replicates and four traps per replicate. The 300 simulated data points (5 Time intervals ×3 Treatments ×5 Replicates ×4 Traps) were generated from a Poisson log-normal distribution. By varying the parameters (mean estimated catch, site variance, trap variance), seven conditions were created for evaluation, with the mean catch being that set for the first treatment (Table 1). The ‘Field Data’ condition parameters were chosen to represent the baseline, i.e. representing the catches found in the field, and then to explore the effects of variation from this in the mean catch (‘Low Mean’ and ‘High Mean’), and in different combinations of replicate site and trap variances (‘Low Site, Low Trap Variances’, etc.) (Table 1). For each of the seven sets of parameters (line entries in Table 1), we simulated 100 datasets.

To create an unbalanced design, these 100 datasets per condition were manipulated to mimic trap loss at a particular activity period of the beetles. We simulated trap loss for two opposing cases: during high beetle activity in high abundance sites (“High catch loss”), and during low beetle activity in low abundance sites (“Low catch loss”) (Table 2). We expected the traditional standardization techniques (see below) to underestimate the number of individuals, as compared to datasets with no losses, in the first case and overestimate in the second, both examples leading to underestimates in the effect size. While other trap loss scenarios could have been used as examples to highlight problems associated with unequal sample sizes, the ones chosen represent two clear cases of situations described in the upper right and lower left panel in Table 2.

We used four levels of loss, randomly ‘losing’ 18.75%, 37.5%, 56.25% or 75% of the traps during the first and second time intervals in Treatments 2 and 3 for the high catch case, and the same percentages during the fourth and fifth time intervals in Treatments 1 and 2 (Table 2). This equates to 15, 30, 45 and 60 of the 80 data points lost from these four cells (2 Intervals ×2 Treatments), resulting in five situations for comparison; full datasets (no losses), and datasets 5% Loss, 10% Loss, 15% Loss and 20% Loss. These percentages refer to the % number of traps lost in the whole design.

The following five analyses were performed on these datasets;

*1. Traditional standardization, Normal model*. This procedure is common in the carabid beetle literature [26], [30], see also [31]. The catch is usually standardized to 100 trapping days, and analyzed at the replicate level (i.e. summed over the four traps and the five time intervals). In our worked example, each replicate was expected to be actively collecting beetles for 400 trap days over the season (4 traps ×20 days per time interval ×5 time intervals). The total catch of a replicate was standardized to 100 days by dividing the catch by the number of trap days (400 if no traps were lost) and multiplying by 100. In the event of losing a trap from a replicate, the total catch of the replicate was corrected (i.e. standardized) by dividing by the number of trap days of the traps that were recovered (i.e. not lost) and multiplying by 100. As is evident from this procedure, seasonal activity is not taken into account.

This standardization procedure resulted in five values (replicates) per Treatment. The data were log transformed to normalize the errors and the model was simply (see [32] for notation): ln(Abundance+1) ∼ Treatment. The response variable was defined as following a normal (Gaussian) error distribution and the Treatment factor had three levels. Predictions were calculated as log(e*^µ^*-1), where *µ* is the log(expected abundance +1) from the model.

*2. Known seasonality, Normal model*. If seasonal activity throughout the trapping period is known (from *a priori* knowledge, see above), the seasonal activity of the time interval and the number of traps operational during each time interval can be included into the model as offset terms. An offset is a term to be added to a linear predictor, such as in a generalized linear model, with a known coefficient ‘1′ rather than an estimated coefficient. Here again, the data were log transformed to approach approximate normality, and because of this transformation, we log transformed the offsets to gain a relationship where, statistically, the doubling of an offset variable value resulted in the doubling in the predicted catch (calculated from the model). The model in R was: ln(Abundance +1) ∼ Treatment + offset(ln(percent per Time interval)) + offset(ln(no. traps)). The response variable was defined as following a normal (Gaussian) error distribution and the Treatment factor had three levels. The percent per Time interval was the expected catch percentages from *a priori* knowledge of the activity of *P. oblongopunctatus* (25%, 49%, 13%, 9% and 4%) and no. traps was 4 if none of the four traps per replicate per time interval was lost, 3 if one of the traps was lost, etc.

*3. Unknown seasonality, Normal model*. For many species the activity throughout the observation period is not known. When this is the case, the seasonality term in the models can be added as a factor – in our example with five levels (5 time intervals). The model in R was: ln(Abundance +1) ∼ Treatment + Time interval + offset(ln(no. traps)). The response variable was modeled following a normal distribution and the Treatment factor had three levels. Time interval was a fixed effect factor with five levels (resulting from five visits to empty the traps), and no. traps was as above.

*4. Known seasonality, Negative Binomial model*. This procedure was the same as number two above, except that the response variable, Abundance, was modeled following a negative binomial distribution. Note that this is not the same model as was used to generate the data, as the data were simulated from a Poisson log-normal distribution, although there may be little difference between the estimates [19].

*5. Unknown seasonality, Negative Binomial model*. This procedure was the same as number three above, except that the response variable, Abundance, was modeled following a negative binomial distribution.

Model results were compared to the simulated values by calculating the mean bias of the treatment means, on the log scale. These were averaged over the simulations and summed over the three treatments. Ideally the bias should be close to zero: this means that the method will, on average, return the true value, whereas a positive bias would suggest that the method overestimates the effect, and a negative bias suggests underestimation.

All simulations and analyses were carried out in the R statistical program, version 2.9.1 [33], using the MASS [34] and lme4 [35] packages (see Supporting Information S1).

## Results

Our main results are as expected; 1) unequal sample sizes lead to over- or underestimates in the effect size if seasonality is not taken into account in data that have a seasonal pattern, and 2) negative binomial models return more accurate estimates of effect sizes than normal models (Figs. 1, 2) (see also [19]).

Estimates of the means for the simulations based on the field data with losses during high activity periods are plotted in Fig. 1, and with losses during low activity periods in Fig. 2. Trap losses affected all normal models, with biases in both directions and changes in the bias as sample sizes became more unbalanced. The traditional standardization method underestimated the catch when trap losses occurred during high activity periods, and overestimated the catch when trap losses occurred during low activity periods (Figs. 1, 2). In contrast, the negative binomial methods consistently give the same estimate when samples are lost with only a decrease in precision, and have the lowest bias. Generally, there was little effect of unequal sample sizes on variation in the negative binomial model estimates, a pattern that is repeated with the simulations from the other six sets of parameters (see Table 1 and Supporting Information S2). It is thus enough in what follows to examine the mean bias.

The effects of changing the mean estimated catch and variation between sites and traps (see Table 1 for details) are shown in Fig. 3. At low estimated mean catch, the bias is positive and negative for the normal models, while close to zero for the negative binomial models. Bias is low for all models when the mean estimated catch is high. Qualitatively, the effects of changing trap loss were similar for all combinations of site and trap variances used, with the traditional standardization techniques often being highly susceptible to unequal sample sizes. Generally, the negative binomial models have a small negative bias.

## Discussion

The statistical evaluation of data forms an integral part of most quantitative research. Quite alarmingly, however, it seems that a substantial proportion of statistical tests reported in the literature are incorrectly applied [36]. We showed that a standardization technique used frequently in the carabid beetle literature [30], [37], as well as in studies on other organisms [31], may seriously bias the estimates of the true treatment effect. Both underestimates and overestimates are possible.

The effects of unequal sample sizes are clear: for example sample losses during high activity in high abundance treatments underestimates the effect size when using the traditional method and the same holds true for losses during low activity in low abundance treatments, as predicted. Logically, overestimates of the effect size can be expected with data losses in low abundance treatments during high detectability periods as well as high abundance treatments during low detectability periods.

We also observed a bias in the negative binomial estimates, particularly when site variation was high. However, this was generally smaller than for the normal models, and not affected by unequal sample size. It is worth noting that the data were simulated using a log-normal distribution, so the fitted model was actually incorrect. Models assuming a quasi-Poisson distribution gave a very similar bias to the negative binomial models (data not shown), but this is not always the case. The key issue here is the relationship between the mean and variance of the distribution, which can be examined as a part of model checking [23]: there will certainly be times when the log-normal provides a better fit to the data.

With an unbalanced design in space and time, the main findings and recommendations of this paper are as follows. If the observation period includes time intervals with different detectability of the focal research object, we recommend that data collected at each time interval should not be merged (see e.g., [38]). When analyzing the data, the researcher has two options: (a) if reliable prior information is available on how detectability of the research object varies (e.g. seasonal activity of the species), the expected percentage of occurrences (e.g. % catch) for each time interval, and the length of the sampling interval (e.g. the number of days a trap was operational during each interval) should be added to the model as offset terms; or (b) if no reliable information is available on the variation in detectability, the time intervals should be added to the model as a factor, and the observational effort during each time interval as an offset term.

If sampling is done simultaneously at different sites and sampling effort is the same (e.g., the design is balanced and no samples are lost) throughout the observation period, seasonal variation is not important when data are analyzed. However, this scenario is quite uncommon. Traps cannot be placed at all sites at exactly the same time, survey sampling cannot be performed simultaneously, and data are lost through unforeseen events. Consequently, an unbiased method is needed to standardize across the data. Here we show that detectability (in our example seasonality), included either as a percentage variable or as a free factor in negative binomial statistical models, considerably improved the accuracy of the results, even with a loss of up to 20% of the data points.

In the example used here, the often-used traditional standardization procedure seriously mis-estimated the effect even without trap loss, and could seriously underestimate the effect size with trap loss. For example, at 20% loss the estimated mean number of beetle individuals collected at Treatment 2 was only 20% higher than that of Treatment 1 (it should have been 100% higher), and for Treatment 3 it was only 60% higher than Treatment 1 (it should have been 400% higher) (Fig. 1).

The distribution of many, if not most biological count data (for example number of individuals) is typically lumpy, with more variation than if everything was random. There are several approaches to incorporating this into models, here we used the negative binomial distribution [18], [39], [40], but other approaches are also possible [23]. We recommend that one of these methods be used to model the variation in response variable when the researcher suspects aggregation of the count variable at the scale of the study [21]. Which method is most appropriate will depend on the form of heteroscedasticity in the data, i.e. how the variance in the residuals changes with the mean.

Often during statistical analyses, scientists either ignore the assumptions of the data (e.g. normality, independent observations and homoscedasticity), transform the data without checking whether the transformation adequately corrected the problem, or use non-parametric tests without realizing that these tests also have various assumptions [18], [41]. The ANOVA procedure is quite robust, even when assumptions of normality and homogeneity of variance are violated considerably, but it seems logically better to use a statistical model that is appropriate for the data being analyzed, such as the negative binomial model for clumped counts data [18].

Most commercial statistical packages include options to define the error distribution as Gaussian, Poisson or negative binomial, as well as others. Little mathematical knowledge is required to run these analyses [32], the ecologist needs to have a basic knowledge of statistics, and the assumptions and workings of various distributions. These details are discussed in many statistical textbooks [42], [43], but are often not implemented. The challenge is to identify, and to correct, flaws in field methods (such as seasonal activity and its effects on population estimates if sampling designs are unbalanced), and to interpret statistical results in a biologically meaningful way.

## Supporting Information

Supporting Information S1 **R code used in this study.**
(PDF)

Supporting Information S2 **Box and whisker plots of the effect sizes (predicted catch) of the analyses performed on the manipulated data (low and high means, low and high treatment site and trap variance, see** **Table 1** **).** The black horizontal lines represent the simulated (i.e. true) total abundances per treatment without trap loss. The x-axis represents the three Treatment levels with five conditions per treatment (from no loss to 20% loss). Figs. S1–S6 are for trap losses at “High catch loss” (see Table 2). Figs. S7–S12 are for trap losses at “Low catch loss” (see Table 2). Figs. S13–S18 are for “Random trap losses”. The last panel in each figure represents the mean bias of the models against trap loss (see Fig. 3 in the manuscript for explanations).
(PDF)
