# Supplementary material for: Dealing with Varying Detection Probability, Unequal Sample Sizes and Clumped Distributions in Count Data
Source: PLoS One. 2012 Jul 20;7(7):e40923. doi: 10.1371/journal.pone.0040923 (PMC3401226; doi:10.1371/journal.pone.0040923)
Supplement: Supporting Information S1 — R code used in this study. (PDF) [file pone.0040923.s001.pdf]

Dealing with Varying Detection Probability, Unequal Sample Sizes and Clumped Distributions in Count Data

D. Johan Kotze*[1], Robert B. O'Hara[2,3] & Susanna Lehvävirta[1,4]

[1] *Department of Environmental Sciences, PO Box 65, FI-00014, University of Helsinki, Finland;* [2] *Department of Mathematics and Statistics, PO Box 68, FI-00014, University of Helsinki, Finland;* [3] *Biodiversity and Climate Research Centre, Senckenberganlage 25, D-60325 Frankfurt am Main, Germany;* [4] *Botanic Garden, Finnish Museum of Natural History, PO Box 44, FI-00014, University of Helsinki, Finland*

Supporting Information S1. R code used in this study.

```
###############################################################
###                                                         ###
###   This is all the R code for the simulations            ###
###            and output in the paper                      ###
###                                                         ###
###          Code written by Bob O'Hara                     ###
###                                                         ###
###############################################################

# The functions are provided "as is", and can be used freely.
# The only restriction is that I want Greenland, Svalbard and Tahiti
# if you use the code to help you conquor the world.

# First up are a pile of functions, that are used later.
# Below them is the code to do the simulations and create the output.
# Search for Thweseld to get there
# You should just be able to run the code "as is",
# but set the working directory so that you know where the output is!
# To do that change the directory here:

setwd("~/Dropbox/TrapLoss")

# With the directory where you want the output between the quotes.
# The code will create a folder there called Data/,
# unless there is one there already.  It will write a pile of files there too

###############################################################
###############################################################
###                                                         ###
###                                                         ###
###                                                         ###
###     All the Functions you need                          ###
###                                                         ###
###                                                         ###
###                                                         ###
###############################################################
###############################################################

###############################################################
#### Log-normal simulation of one data set

# Arguments
# Treat.Mean - Mean abundace for each treatment, over whole season
```

```
# Proportion.Season - proportion of catch in each season
# NRep - number of replicate plots
# NTrap - number of traps per replicate plot
# sdRep - standard deviation in Rep effect (log-normally distributed)
# sdTrap - standard deviation in Trap effect (log-normally distributed)

# Value
# Data frame with the data in it, with the following variables:
# Treatment - Which treatment
# Rep  - Which rep (nested within treatment)
# Season - Which season
# Prop.Season - True proportion of catch for that season
# Trap -. Which trap
# TrueMean - True mean abundance
# Count - Number of individuals "caught"

SimBeetlesLN=function(Treat.Mean, Proportion.Season, NRep, NTrap, sdRep, sdTrap)
 {
# NRep= number of replicate sites per treatment
  NTreat=length(Treat.Mean)
  NSeason=length(Proportion.Season)

  Design=expand.grid(Trap=1:NTrap, Rep=1:NRep, Season=1:NSeason, Treatment=1:NTreat)
  Design$Prop.Season=Proportion.Season[Design$Season]
  Design$Rate.Treat=log(Treat.Mean[Design$Treatment])   # Put everything on log scale
  Design$RepMean=apply(Design, 1, function(df, Mu) { Mu[df["Season"], df["Treatment"]] },
                         Mu=matrix(rnorm(NTreat*NRep, -(sdRep^2)/2,sdRep), nrow=NSeason) )
#                         Mu=matrix(rnorm(NTreat*NRep, 0,sdRep), nrow=NSeason) )


  Design$Trap.Rand=rnorm(dim(Design)[1], -(sdTrap^2)/2,sdTrap)
#  Design$Trap.Rand=rnorm(dim(Design)[1], 0,sdTrap)


  Design$TrueMean=exp(Design$Rate.Treat + Design$RepMean + Design$Trap.Rand +
log(Design$Prop.Season))
  Design$Count=rpois(dim(Design)[1], Design$TrueMean)

  Data=Design[,c("Treatment", "Rep", "Season", "Prop.Season", "Trap", "TrueMean", "Count")]
  return(Data)
}

################################################################
# Function to simulate data

# largely a wrapper for SimBeetlesLN, but adds vectors showing which traps to remove
# Arguments
# set - object containing the parameters to use
# LoseProps - proportion of traps lost in each season
# Prop.S - True proportion of catch for that season
# NRep - number of replicate plots
# NTrap - number of traps per replicate plot
# NSims - number of simulated data sets to create, deaults to 100
# FileName - Strem for name of files to create.  Defaults to "Data/SimData"

# Value
# Doesn't return anything, but quietly saves NSims data sets
# The file names default to "Data/SimDataX.dat", where X is the index of the simulation
# The files contain a data frame called Data produced by SimBeetlesLN(),
# but augmented with 5 columns of 0s and 1s:1 indicates the trap was "lost"
# Remove0 - No loss
# Remove5 - 5% trap loss
# Remove10 - 10% trap loss
# Remove15 - 15% trap loss
# Remove20 - 20% trap loss

DataSimulations=function(Parameters, LoseProps, Prop.S, NRep, NTrap, NSims=100,
FileName="Data/SimData") {
```

```
  STEM=paste(FileName, gsub("Pars","",Parameters$Name), sep="")
  for(i in 1:NSims) {
    Data=SimBeetlesLN(Parameters$Tr.mu, Prop.S, NRep, NTrap, Parameters$sdRep,
Parameters$sdTrap)
    Data$Remove0=rep(0, dim(Data)[1])
    Data$Remove5=RemoveInd(Data, LoseProps$Prop.Lose05)
    Data$Remove10=RemoveInd(Data, LoseProps$Prop.Lose10)
    Data$Remove15=RemoveInd(Data, LoseProps$Prop.Lose15)
    Data$Remove20=RemoveInd(Data, LoseProps$Prop.Lose20)
    save(Data, file=paste(STEM,i,".dat",sep=""))
  }
}

###############################################################
# Function to create indices for observations to remove for reduced data.

# Arguments
# df - data frame with Treatment and Season
# Prop - matrix containing probability for each trap to be lost in that season & treatment
combination

# Value
# Binary vector: 1 = trap was lost

RemoveInd=function(df, Prop) {
  apply(cbind(df$Treatment, df$Season), 1, function(xx, Pr)
  return(rbinom(1,1,Pr[xx[1], xx[2]])), Pr=Prop)
}

###############################################################
# Function to run the analyses on all the data sets.  Essentially a wrapper +
# formatting for GetResults()
# Outputs results into .RData file

# Arguments

# set - parameter set
# Pars - Sets of parameters used to create the data.
# inds - Indictor for which simulated data (typically 1:NSim)
# inbase - prefix for filename(s) where the data are
# outbase- prefix for filename(s) to output results to,  Defaults to "Data/Results"
# add - value to add to counts for log-normal analyses.
# ProportionalSeason - vector of true proportions of the catch in a season.

# Value
# nothing, but naughtily saves the results in a file, in "Data/ResultsX.RData", where X is the
# the file contains an object called Results, with the following:
# TruePars - true parameters
# TradGaus - Matrix with results from traditional Gaussian model (see GetResults() for
details)
# KnownSGaussian - Matrix with results from Gaussian model with known proportion of catch(see
GetResults() for details)
# UnknownSGaussian - Matrix with results from Gaussian model without known proportion of
catch(see GetResults() for details)
# KnownSNegBin - Matrix with results from Negative Binomial model with known proportion of
catch(see GetResults() for details)
# UnknownSNegBin - Matrix with results from Negative Binomial model without known proportion
of catch(see GetResults() for details)

RunAnalyses=function(set, Pars, inds, inbase, outbase="Data/Results", add=1,
ProportionalSeason=NULL) {
  stem=paste(inbase, gsub("Pars","",set), sep="")

  TradGaus=lapply(paste("Remove",c(0,5,10,15,20),sep=""), function(Rem, Inds)
    sapply(Inds, GetResults, remove=Rem, type="TradGaus", STEM=stem, ADD=add,
truepars=Pars[[set]]$Tr.mu), Inds=inds)
    names(TradGaus)=paste("Remove",c(0,5,10,15,20),".",sep="")
```

```
  KnownSGaussian=lapply(paste("Remove",c(0,5,10,15,20),sep=""), function(Rem, Inds)
    sapply(Inds, GetResults, remove=Rem, type="KnownSGaussian", STEM=stem, ADD=add,
PropS=ProportionalSeason, truepars=Pars[[set]]$Tr.mu),
                Inds=inds)
    names(KnownSGaussian)=paste("Remove",c(0,5,10,15,20),".",sep="")
  UnknownSGaussian=lapply(paste("Remove",c(0,5,10,15,20),sep=""), function(Rem, Inds)
     sapply(Inds, GetResults, remove=Rem, type="UnknownSGaussian", STEM=stem, ADD=add,
truepars=Pars[[set]]$Tr.mu), Inds=inds)
    names(UnknownSGaussian)=paste("Remove",c(0,5,10,15,20),".",sep="")
  KnownSNegBin=lapply(paste("Remove",c(0,5,10,15,20),sep=""), function(Rem, Inds)
     sapply(Inds, GetResults, remove=Rem, type="KnownSNegBin", STEM=stem,
PropS=ProportionalSeason, truepars=Pars[[set]]$Tr.mu), Inds=inds)
    names(KnownSNegBin)=paste("Remove",c(0,5,10,15,20),".",sep="")
  UnknownSNegBin=lapply(paste("Remove",c(0,5,10,15,20),sep=""), function(Rem, Inds)
     sapply(Inds, GetResults, remove=Rem, type="UnknownSNegBin", STEM=stem,
truepars=Pars[[set]]$Tr.mu), Inds=inds)
    names(UnknownSNegBin)=paste("Remove",c(0,5,10,15,20),".",sep="")

  Results=list(
    TruePars=Pars[[set]],
    TradGaus=TradGaus,
    KnownSGaussian=KnownSGaussian,
    UnknownSGaussian=UnknownSGaussian,
    KnownSNegBin=KnownSNegBin,
    UnknownSNegBin=UnknownSNegBin
  )
  save(Results, file=paste(outbase, set, ".RData", sep=""))
}

###############################################################
# Function to estimate the statistics for a single data set
# Can chose models (incl. quasi-Poison, whose results are not reported in the paper

# Arguments
# ind - Indictor for which simulated data (typically 1:NSim)
# remove - Vector for which data points (traps) to remove 1=remove.
# type - Which estimation method to use.  Possible values:
#            TradGaus - traditional method
#            KnownSGaussian - (log-)Normal, with known seasonality
#            UnknownSGaussian - (log-)Normal, with unknown seasonality
#            KnownSNegBin - Negative binomial, with known seasonality
#            UnknownSNegBin - negative binomial, with unknown seasonality

#            KnownSQPois - Quasi-Poisson, with known seasonality
#            UnknownSQPois - Quasi-Poisson, with known seasonality
#
# STEM - prefix for name of data, used as input.  Defaults to"Data/SimData"
# ADD - value to add to counts before log transformation.  Defaults to 0.1
# PropS - vector of true proprtions of catch per season.
# truepars - true values of parameters (for estimation of bias & RMSE)

# Value
# Vector with the following:
# p.treat - p-value for test of a treament effect (from anova())
# Coefs - Coefficients for each tratment: one per treatment
# MeanBias - Mean bias
# RMSE - Root Mean Squared Error

GetResults=function(ind, remove, type, STEM="Data/SimData", ADD=1, MinPred=-5, PropS=NULL,
truepars) {
  require(MASS)

  load(paste(STEM,ind,".dat",sep=""))
  LossData=subset(Data, !Data[,remove])
# Create aggregated data for old method
  data=AggData(LossData, KeepSeason=!(type=="TradGaus"), SeasonProp=PropS)
  lm1=switch(type,
# Treat: for whole season, for 100 trap days in treatment
```

```
        TradGaus = lm(log(100*x/Trapdays+ADD)~Treat-1, data=data),
# Treat: for whole season (Prop.Season=1), for 1 site, for 1 trap
        KnownSGaussian = lm(log(x+ADD)~Treat+offset(log(Prop.Season))+offset(log(No.Traps))-1,
data=data),
# Treat: for one season, for 1 site, for season 1, for 1 trap
        UnknownSGaussian=lm(log(x+ADD)~Treat+Season+offset(log(No.Traps))-1, data=data),
        KnownSNegBin=glm.nb(x~Treat+offset(log(Prop.Season))+offset(log(No.Traps))-1,
data=data),
        UnknownSNegBin=glm.nb(x~Treat+Season+offset(log(No.Traps))-1, data=data),

        KnownSQPois=glm(x~Treat+offset(log(Prop.Season))+offset(log(No.Traps))-1, data=data,
family="quasipoisson"),
        UnknownSQPois=glm(x~Treat+Season+offset(log(No.Traps))-1, data=data,
family="quasipoisson")

  )

  pval=switch(type,
        TradGaus = anova(lm1, test="F")$'Pr(>F)'[1],
        KnownSGaussian = anova(lm1, test="F")$'Pr(>F)'[1],
        UnknownSGaussian=anova(lm1, test="F")$'Pr(>F)'[1],
        KnownSNegBin=anova(lm1)$'P(>|Chi|)'[2],
        UnknownSNegBin=anova(lm1)$'P(>|Chi|)'[2],

        KnownSQPois=anova(lm1)$'P(>|Chi|)'[2],
        UnknownSQPois=anova(lm1)$'P(>|Chi|)'[2]
  )
  Coefs=coef(lm1)[grep("Treat", names(coef(lm1)))]  # On log scale

# Correct to total over seasons: calculate mean season, and add log(no of seasons)
  if(length(grep("Unknown",type, fixed=T))>0) {
    Season=c(0, coef(lm1)[grep("Season", names(coef(lm1)))])
    Coefs=Coefs + log(sum(exp(Season)))
  }
  CNames=names(Coefs)
  if(grepl("Gaus", type)) Coefs=log(pmax(exp(MinPred),exp(Coefs)-ADD))
  names(Coefs)=CNames

  return(c(
             p.treat=pval,
             Coefs=Coefs,
# On log scale
             MeanBias=mean(Coefs-log(truepars)),
             RMSE=mean((Coefs-log(truepars))^2)
  ))
}

################################################################
# Function to aggregate data from a simulation, by trap and by season
# if KeepSeason=F is given

# Arguments
# df - data frame with, well, data in it.  Should include Count, Tratment, and Rep
# KeepSeason - Should counts NOT be summed over season?  Defaults to TRUE (i.e. keep the
season)
# SeasonProp - proporion of catch per season (this is just passed through the function)

# Value
#    Data frame with ...
# Treatment - treatment
# Rep - replicate
# Count (summed over everything else
# No.Traps - Number of traps in each sum
# TrueMean - True expectec no. of individuals
# Trapdays - number of trap dayes (assuming 20 days per trap) =No.Traps*20
#   And optionally, if KeepSeason is TRUE
# Prop.Season - proportion of the true abundance expeced in the season that trap was used
# Season - The season
```

```
AggData=function(df, KeepSeason=TRUE, SeasonProp=NULL) {
# Set up variables to NOT sum over
  Lst=list(Treat=df$Treatment, Rep=df$Rep)
  if(KeepSeason) Lst$Season=df$Season
  dat=aggregate(df$Count, Lst, sum)
  dat$No.Traps=aggregate(df$Count, Lst,  length)$x  # Number of traps in each sum:
  dat$True=aggregate(df$TrueMean, Lst,  sum)$x  # True expectec no. of individuals

  if(KeepSeason)  {
     dat$Prop.Season=SeasonProp[dat$Season]    # need to add this
     dat$Season=factor(dat$Season)
  }
  dat$Trapdays=dat$No.Traps*20
  dat$Treat=factor(dat$Treat)
  dat$Rep=factor(dat$Rep)
  return(dat)
}

###############################################################
# Function to extract coefficients from a list with the results in it

# Arguments
# list - list, one of those created in RunAnalyes

# Value
# Data frame, with
# Treament: Which treatment
# Coef - Estimated coefficients for that treatment
# Removed - proportion of traps removed (well, expected proportion)

GetCoefs=function(list) {
  ExtractCoefs=function(mat) {
    coefs=mat[grep("Coefs", rownames(mat)),]
    treatment=rep(as.numeric(gsub('[a-zA-Z.]',"", attr(coefs, "dimnames")[1][[1]])) ,
times=dim(coefs)[2])
    cbind(treatment, c(coefs))
  }
  ExtractedCoefs=lapply(list, ExtractCoefs)

  Coefs.df=as.data.frame(cbind(
         sapply(1:2, function(ind, lst) {
           c(unlist(lapply(lst, function(List, IND) List[,IND], IND=ind)))
         }, lst=ExtractedCoefs)
       ))
  colnames(Coefs.df)=c("Treatment", "Coef")

  Coefs.df$Removed=as.numeric(sapply(gsub("Remove","",dimnames(Coefs.df)[1][[1]]),
function(str) {
    strsplit(str, "\\.")[[1]][1]
  }))
  Coefs.df
}

###############################################################
# Function to plot coefficients extracted by GetCoefs

# Arguments
# coefs - data frame with Coef=coefficients, Removed & Treatment are factors
# True - Vector of true coefficients
# Name - Title of plot
# ... - optional arguments passed to boxplot()

# Value
# Nothing, but gives a pretty plot of the coefficients and their true values.

PlotCoefs=function(coefs, True, Name, AddOne=FALSE, Removes=rep(seq(0,20,by=10),3), ...) {
  at.plot=rep(1:5,3)+rep(c(0,6,12),each=5)
```

```
  Line.start=c(0.6,6.6,12.6)
  Coefficients=exp(coefs$Coef)-as.numeric(AddOne)
  boxplot(Coefficients~coefs$Removed*coefs$Treatment,
             xaxt="n", at=at.plot, xlim=c(0.9,17.1), border="grey40",  ...)
    segments(Line.start, True, Line.start+4.8, True, lwd=2, lty=1)
    axis(1, Removes, at=rep(c(1,3,5),3)+rep(c(0,6,12),each=3), cex.axis=1)
    title(main=Name)
}

###############################################################
# Function to plot all parameter sets

# Arguments
# Results - List, structure as that saved by RunAnalyses() (see above)
# Names - Vector of names, passed to PlotCoefs

# Value
# Nothing, but plots up to 6 graphs of parameters.

PlotAllCoefs=function(Results, Bias, Names, BiasSym=NULL, removals="") {
  MaxY=exp(max(sapply(2:length(Results), function(ind, List) {
     max(GetCoefs(List[[ind]])$Coef)
  }, List=Results)))

  removes=rep(seq(0,20,by=10),3)
  if(removals=="High") removes=removes*rep(c(0,1), c(3,6))
  if(removals=="Low") removes=removes*rep(c(1,0), c(6,3))
  par(mfrow=c(2,3), mar=c(3,2,4,1), las=1, oma=c(2,3,0,1))
  sapply(2:length(Results), function(ind, List, true, Names, maxY) {
    Res=GetCoefs(List[[ind]])
    PlotCoefs(coefs=Res, True=true, Name=Names[ind-1], AddOne=as.logical(length(grep("Gaus", 
Names[ind-1]))), ylim=c(0, maxY), Removes=removes)
  },List=Results, true=Results[[1]]$Tr.mu, Names=Names, maxY=MaxY)

  Removed=as.numeric(gsub('\\.', "",gsub('Remove', "",rownames(Bias))))
  plot(Removed, Bias[,1], type="n", ylim=range(Bias))
  sapply(1:dim(Bias)[2], function(ind, df,xx, SYM=NULL) {
     if(is.null(SYM)) SYM=1:dim(df)[1]
     lines(xx, df[,ind])
     points(xx, df[,ind], pch=SYM[ind])
  }, df=Bias,xx=Removed, SYM=BiasSym)
  abline(h=0, lty=3)

  mtext("Percentage of traps lost", 1, outer=T)
  mtext("Estimated Abundance",2, outer=T, line=0.5, las=0)
}

###############################################################
# Function to extract biases

# Arguments
# ParSet - List, structure as that saved by RunAnalyses() (see above)
GetMeanBias=function(ParSet) {
    TrueMean=log(ParSet[[1]]$Tr.mu)
    as.data.frame(lapply(ParSet[-1], function(EstMeth) { # Over est. methods
      unlist(lapply(EstMeth, function(Rems) mean(Rems["MeanBias",]) ))
    }))
}

###############################################################
# Function to plot biases

# Arguments
# Sets - Vector with names of parameter sets for which the plots are wanted
# Bias - list, with biases in them

# Value
# Nothing, but plots a nice graph of the biases against the proportion of traps removed.
```

```
PlotBias=function(Sets, Bias, Names=NULL, ...) sapply(Sets, function(set, Res,symbol, names,
...) {
  Bias=Res[[set]]
  Removed=as.numeric(gsub('\\.', "",gsub('Remove', "",rownames(Bias))))
  plot(Removed, Bias[,1], type="n", ...)
mtext(names[set], 4, line=0.2, padj=1)
  sapply(1:dim(Bias)[2], function(ind, df,xx, SYM=1:dim(df)[1]) {
     lines(xx, df[,ind])
     points(xx, df[,ind], pch=SYM[ind])
  }, df=Bias,xx=Removed, SYM=symbol)
  abline(h=0,lty=3)
}, Res=Bias, names=Names, ...)

# Sorry, no more.

################################################################
###                                                          ###
###   The code to run the simulations.                       ###
###                                                          ###
###   It requites a lot of functions, which are all          ###
###   after this code: scroll down to find it.               ###
###                                                          ###
###   If you want to use this a few times, copy the          ###
###   functions into a new file, and use source(FILENAME)    ###
###   to read the functions in the file (called FILENAME)    ###
###                                                          ###
###              Thweseld                                    ###
###                                                          ###
################################################################

# Some setting up of Stuff

# Set the directory where you want to work: the figured will appear in this
# file, and the data in a Data/ subdirectory.
# you might have already set this, but just in case...
# setwd("~/Dropbox/TrapLoss")
# source("SupplInfoApp1Functions.R")

library(parallel)

# Check to see if there is a directory called Data.  If not, it creates one.
# If there is a file called Data, you will get a warning.
if(is.na(file.info("Data")[[2]])) {
  cat("Warning: no Data/ directory.  Creating one.\n")
  dir.create("Data")
} else {
  if(!file.info("Data")[[2]]) cat("Data is not a directory: please rename it (for the moment),
and create a directory\n")
}

################
# Create simulated data

# Parameters, taken from real data
POblResults=list(sdResid=0.4873454, sdSite=0.1433374, sdTrap=0.2999971, LowestMean=1.472805)

# Number of data sets simulated per parameter set
NSims=100

# Parameter sets

# From the data
DataPars=list(sdRep=POblResults$sdSite, sdTrap=POblResults$sdTrap,
Tr.mu=POblResults$LowestMean*c(1,2,4))

# Set up factorial model for each combination of levels
ParSets=expand.grid(
```

```
  sdRep=c("L","O","H"),
  sdTrap=c("L","O","H"),
  Tr.mu=c("L","O","H"),
    stringsAsFactors = F
)
ParSetsChar=apply(ParSets,1,function(char) paste(char[1],char[2],char[3],sep=""))

SimSets=sapply(ParSetsChar,function(set, true) {
  setNum=sapply(strsplit(set, NULL)[[1]],switch, L = 1/5, O = 1, H = 5)
  Pars=list(Name=set, sdRep=true$sdRep*setNum[1], sdTrap=true$sdTrap*setNum[2],
Tr.mu=true$Tr.mu*setNum[3])
#  assign(paste("Pars",set[1],set[2],set[3],sep=""), Pars)
  Pars
}, true=DataPars, simplify=F)

# Experimental setup
# Proportion of traps to lose from each season
# Which treatment and season: high activity loss
Prop.LoseAll.HighAct=matrix(c(
#   Season 1,  S 2,  S 3,  S 4,  S 5
       0, 0, 0, 0, 0,      # treatment 1
       1, 1, 0, 0, 0,      # treatment 2
       1, 1, 0, 0, 0),     # treatment 3
nrow=3, byrow=T)

# Which treatment and season: low activity loss
Prop.LoseAll.LowAct=matrix(c(
#   Season 1,  S 2,  S 3,  S 4,  S 5
       0, 0, 0, 1, 1,      # treatment 1
       0, 0, 0, 1, 1,      # treatment 2
       0, 0, 0, 0, 0),     # treatment 3
nrow=3, byrow=T)

# Which treatment and season: random activity loss
Prop.LoseAll.Random=matrix(c(
#   Season 1,  S 2,  S 3,  S 4,  S 5
       1, 1, 1, 1, 1,      # treatment 1
       1, 1, 1, 1, 1,      # treatment 2
       1, 1, 1, 1, 1)*(4/15),     # treatment 3
nrow=3, byrow=T)

# Proportions to lose at different levels of loss
LoseProps.HighAct=list(
  Prop.Lose0=Prop.LoseAll.HighAct*0,         # No loss
  Prop.Lose05=Prop.LoseAll.HighAct*0.1875,   # 5% loss
  Prop.Lose10=Prop.LoseAll.HighAct*0.375,    # 10% loss
  Prop.Lose15=Prop.LoseAll.HighAct*0.5625,   # 15% loss
  Prop.Lose20=Prop.LoseAll.HighAct*0.75,     # 20% loss
  Prop.Lose25=Prop.LoseAll.HighAct*(0.75+0.1875)   # 25% loss
)
LoseProps.LowAct=list(
  Prop.Lose0=Prop.LoseAll.LowAct*0,         # No loss
  Prop.Lose05=Prop.LoseAll.LowAct*0.1875,   # 5% loss
  Prop.Lose10=Prop.LoseAll.LowAct*0.375,    # 10% loss
  Prop.Lose15=Prop.LoseAll.LowAct*0.5625,   # 15% loss
  Prop.Lose20=Prop.LoseAll.LowAct*0.75,     # 20% loss
  Prop.Lose25=Prop.LoseAll.LowAct*(0.75+0.1875)   # 25% loss
)
LoseProps.Random=list(
  Prop.Lose0=Prop.LoseAll.Random*0,         # No loss
  Prop.Lose05=Prop.LoseAll.Random*0.1875,   # 5% loss
  Prop.Lose10=Prop.LoseAll.Random*0.375,    # 10% loss
  Prop.Lose15=Prop.LoseAll.Random*0.5625,   # 15% loss
  Prop.Lose20=Prop.LoseAll.Random*0.75,     # 20% loss
  Prop.Lose25=Prop.LoseAll.Random*(0.75+0.1875)   # 25% loss
)

# Simulations of data
```

```
# outputs into Data/ sub-directory.
# Filenames: SimDataHighActX.dat, where X  is the number of the simulation.
# and HighAct, LowAct and Random are for
# high activity trap loss,
# low activity trap loss,
# and random(i.e. uniform over the season), respectively

mclapply(SimSets, DataSimulations, LoseProps=LoseProps.HighAct,
            NRep=5,                     # Number of reps per treatment
            Prop.S=c(0.25, 0.49, 0.13,0.09,0.04),     # Proportion of catch in each season
            NTrap=4,                    # Number of traps
            NSims=NSims,   # No. of simulations
            FileName="Data/SimDataHighAct"
)
mclapply(SimSets, DataSimulations, LoseProps=LoseProps.LowAct,
            NRep=5,                     # Number of reps per treatment
            Prop.S=c(0.25, 0.49, 0.13,0.09,0.04),     # Proportion of catch in each season
            NTrap=4,                    # Number of traps
            NSims=NSims,
            FileName="Data/SimDataLowAct"
)
mclapply(SimSets, DataSimulations, LoseProps=LoseProps.Random,
            NRep=5,                     # Number of reps per treatment
            Prop.S=c(0.25, 0.49, 0.13,0.09,0.04),     # Proportion of catch in each season
            NTrap=4,                    # Number of traps
            NSims=NSims,
            FileName="Data/SimDataRandom"
)

######################################################################
# Fit models the models to the simulated data
# Loop over the sets of parameters, and in the function, over the simulated data sets
# Outputs results into Data/ directory in (e.g.) ResultsHighActX.RData, where X=simulation
number

# Known proportion of catch in each season
Prop.S=c(0.25, 0.49, 0.13,0.09,0.04)

# High activity trap loss
mclapply(names(SimSets), RunAnalyses, inds=1:NSims, Pars=SimSets,
inbase="Data/SimDataHighAct", outbase="Data/ResultsHighAct", ProportionalSeason=Prop.S, add=1)
# Low activity trap loss
mclapply(names(SimSets), RunAnalyses, inds=1:NSims, Pars=SimSets,
inbase="Data/SimDataLowAct", outbase="Data/ResultsLowAct", ProportionalSeason=Prop.S, add=1)
# Random trap loss
mclapply(names(SimSets), RunAnalyses, inds=1:NSims, Pars=SimSets,
inbase="Data/SimDataRandom", outbase="Data/ResultsRandom", ProportionalSeason=Prop.S, add=1)

########################################################
# Analyse the results: lots of fiddly stuff for graphs in here!

# Some stuff that's needed for the plots
# Names of the estimation methods
EstNames=c("Traditional\nNormal model", "Known seasonality\nNormal model",
         "Unknown seasonality\nNormal model",
   "Known seasonality\nNegative Binomial", "Unknown seasonality\nNegative Binomial")
# Shorter version
EstNames2=c("Trad., Normal", "Known Seas., Normal", "Unknown Seas., Normal",
   "Known Seas., Neg. Bin.", "Unknown Seas., Neg. Bin.")
# Symbol codes used in plots of bias
EstSymbols=c(4,5,2,18,17)

# Parameter set names
SetNames=c("Original Parameters",   "Low Site, Low Trap variance", "Low Site, High Trap
variance",
  "High Site, Low Trap variance", "High Site, High Trap variance", "Low Mean", "High Mean")

# Parameter sets in original analysis
```

```
OrigSimSets=c("OOO","LLO", "LHO", "HLO", "HHO", "LOO","HOO", "OOL", "OOH")

##########################################
# Read in results, and extract mean biases

Res.LowAct=sapply(OrigSimSets, function(set) {
    load(paste("Data/ResultsLowAct", set, ".RData", sep=""))
    Results
  }, simplify=F)
# Extract the mean bias
MeanBias.LowAct=lapply(Res.LowAct, GetMeanBias)

Res.HighAct=sapply(OrigSimSets, function(set) {
    load(paste("Data/ResultsHighAct", set, ".RData", sep=""))
    Results
  }, simplify=F)
MeanBias.HighAct=lapply(Res.HighAct, GetMeanBias)

Res.Random=sapply(OrigSimSets, function(set) {
    load(paste("Data/ResultsRandom", set, ".RData", sep=""))
    Results
  }, simplify=F)
MeanBias.Random=lapply(Res.Random, GetMeanBias)

# Dig out estimates for orig data
# There are lists of lists in here, so this should help anyone foolish enough
# to try and extract any statistics
Orig.HighAct=Res.HighAct[[1]][[2]]

lapply(Orig.HighAct, function(Rems) {  # Over removals
  apply(Rems[grep("Coefs",rownames(Rems)),],1,mean)
})

#############################################################
####  Fig. 1, plus plots for all parameter sets

# Plot For Parameters From Field Data
# Get the largest estimated coefficient, to use in the plot
MaxY=exp(max(sapply(2:length(Res.HighAct[["OOO"]]), function(ind, List) {
  max(GetCoefs(List[[ind]])$Coef)
}, List=Res.HighAct[["OOO"]])))

# Figure 1
png("Fig1.png", width=540)
#pdf("Fig1.pdf", paper="special", width=8.5, height=6)
#postscript("Fig1.eps", paper="special", width=8.5, height=6, horizontal=F)
  PlotAllCoefs(Res.HighAct[["OOO"]], MeanBias.HighAct[["OOO"]], Names=EstNames, 
BiasSym=EstSymbols, removals="High")
#  legend(10, 0.2, EstNames, pch=EstSymbols, cex=0.6, bg="lightgreen")
dev.off()

# Figure 2
png("Fig2.png", width=540)
#pdf("Fig2.pdf", paper="special", width=8.5, height=6)
#postscript("Fig2.eps", paper="special", width=8.5, height=6, horizontal=F)
  PlotAllCoefs(Res.LowAct[["OOO"]], MeanBias.LowAct[["OOO"]], Names=EstNames, removals="Low", 
BiasSym=EstSymbols)
#  legend(2, 0.2, EstNames, pch=EstSymbols, cex=0.8, bg="lightgreen")
dev.off()

png("Fig1andahalf.png", width=540)
#pdf("Fig1andahalf.pdf", paper="special", width=8.5, height=6)
#postscript("Fig1andahalf.eps", paper="special", width=8.5, height=6, horizontal=F)
  PlotAllCoefs(Res.Random[["OOO"]], MeanBias.Random[["OOO"]], Names=EstNames, 
BiasSym=EstSymbols)
#  legend(10, 0.2, EstNames, pch=EstSymbols, cex=0.6, bg="lightgreen")
dev.off()
```

```
# Plot all estimates in one big file
OrderofPlots=c(6,7,2:5) # low mean, high mean, variances

pdf(file="SupplAppendix.pdf", width=8)
  sapply(1:6, function(ind, set, names, Res, MeanBias, Order, PtSym) {
     meanB=MeanBias[[set[ Order[ind] ]]]
     PlotAllCoefs(Res[[set[ Order[ind] ]]], meanB, Names=EstNames, BiasSym=PtSym,
removals="High")
     mtext(paste("Figure S",ind,". High Catch Loss; ", names[Order[ind]],sep=""), 3, outer=T,
cex=1.2, line=0.6)
  Leg.Y=min(meanB) + 0.25*(max(meanB)-min(meanB))
  if(ind==1) legend(0,Leg.Y, EstNames2, pch=PtSym, cex=0.6, bg="white")
# legend(10,Leg.Y, EstNames2, pch=c(4, 5, 2, 18, 17), cex=0.6, bg="white")
  }, set=OrigSimSets, names=SetNames, Res=Res.HighAct, MeanBias=MeanBias.HighAct,
Order=OrderofPlots, PtSym=c(4, 5, 2, 18, 17))

  sapply(1:6, function(ind, set, names, Res, MeanBias, Order, PtSym) {
     meanB=MeanBias[[set[ Order[ind] ]]]
     PlotAllCoefs(Res[[set[ Order[ind] ]]], meanB, Names=EstNames, BiasSym=PtSym,
removals="Low")
     mtext(paste("Figure S",ind+6,". Low Catch Loss; ", names[Order[ind]],sep=""), 3, outer=T,
cex=1.2, line=0.6)
  Leg.Y=min(meanB) + 0.85*(max(meanB)-min(meanB))
#  legend(10,Leg.Y, EstNames2, pch=c(4, 5, 2, 18, 17), cex=0.6, bg="white")
  }, set=OrigSimSets, names=SetNames, Res=Res.LowAct, MeanBias=MeanBias.LowAct,
Order=OrderofPlots, PtSym=c(4, 5, 2, 18, 17))

  sapply(1:6, function(ind, set, names, Res, MeanBias, Order, PtSym) {
     meanB=MeanBias[[set[ Order[ind] ]]]
     PlotAllCoefs(Res[[set[ Order[ind] ]]], MeanBias[[set[ Order[ind] ]]], BiasSym=PtSym,
Names=EstNames)
     mtext(paste("Figure S",ind+12,". Random Loss; ", names[Order[ind]],sep=""), 3, outer=T,
cex=1.2, line=0.6)
  Leg.Y=min(meanB) + 0.75*(max(meanB)-min(meanB))
#  legend(10,Leg.Y, EstNames2, pch=c(4, 5, 2, 18, 17), cex=0.6, bg="white")
  }, set=OrigSimSets, names=SetNames, Res=Res.Random, MeanBias=MeanBias.Random,
Order=OrderofPlots, PtSym=c(4, 5, 2, 18, 17))
dev.off()

##################################################
# Figure 3: Plot Means and variances
UseSets=c("OOL", "OOH", "LLO", "LHO", "HLO", "HHO") # The parameter set results to use
UseNames=c("Low Mean", "High Mean", "Low Site\nLow Trap", "Low Site\nHigh Trap",
         "High Site\nLow Trap", "High Site\nHigh Trap")
names(UseNames)=UseSets

Ylim=range(c(MeanBias.HighAct, MeanBias.LowAct))

png("Fig3.png", width=400, height=900)
#pdf("Fig3.pdf", paper="special", width=5, height=11)
#postscript("Fig3.eps", paper="special", width=5, height=11, horizontal=F)
par(mfcol=c(6,2), mar=c(2,2,2,1), oma=c(2,2.5,2,3))
PlotBias(Sets=UseSets, Bias=MeanBias.HighAct,symbol=EstSymbols,
         ylim=Ylim, ann=F)
  legend(0.0,1.15,EstNames2, pch=EstSymbols, cex=0.7)
PlotBias(Sets=UseSets, Bias=MeanBias.LowAct, symbol=EstSymbols, Names=UseNames,
         ylim=Ylim, ann=F)
  mtext("Percentage of traps lost", 1, outer=T, line=1)
  mtext("Bias", 2, outer=T, las=0, line=0.5)

  mtext("High Catch Loss", 3, outer=T, las=0, at=0.77) #, col="white")
  mtext("Low Catch Loss", 3, outer=T, las=0, at=0.28)

dev.off()

###########################################################################
# That's all folks!
```
