# Supplementary material for: Dealing with Varying Detection Probability, Unequal Sample Sizes and Clumped Distributions in Count Data
Source: PLoS One. 2012 Jul 20;7(7):e40923. doi: 10.1371/journal.pone.0040923 (PMC3401226; doi:10.1371/journal.pone.0040923)

Dealing with Varying Detection Probability, Unequal Sample Sizes and Clumped Distributions in Count Data

D. Johan Kotze*[1], Robert B. O'Hara[2,3] & Susanna Lehvävirta[1,4]

[1] *Department of Environmental Sciences, PO Box 65, FI-00014, University of Helsinki, Finland;* [2] *Department of Mathematics and Statistics, PO Box 68, FI-00014, University of Helsinki, Finland;* [3] *Biodiversity and Climate Research Centre, Senckenberganlage 25, D-60325 Frankfurt am Main, Germany;* [4] *Botanic Garden, Finnish Museum of Natural History, PO Box 44, FI-00014, University of Helsinki, Finland*

Supporting Information S2. Box and whisker plots of the effect sizes (predicted catch) of the analyses performed on the manipulated data (low and high means, low and high treatment site and trap variance, see Table 1). The black horizontal lines represent the simulated (i.e. true) total abundances per treatment without trap loss. The x-axis represents the three Treatment levels with five conditions per treatment (from no loss to 20% loss). Figs. S1 – S6 are for trap losses at "High catch loss" (upper right box in Table 2). Figs. S7 – S12 are for trap losses at "Low catch loss" (lower left box in Table 2). Figs. S13 – S18 are for "Random trap losses". The last panel in each figure represents the mean bias of the models against trap loss (see Fig. 3 in the manuscript for explanations).

-----"High catch loss"-----

Figure S1. **Low mean** beetle catch: predicted effect sizes with trap loss at Treatments and Trapping Intervals of high beetle activity. Replicate site and trap variance from the field data.

Figure S2. **High mean** beetle catch: predicted effect sizes with trap loss at Treatments and Trapping Intervals of high beetle activity. Replicate site and trap variance from the field data.

Figure S3. **Mean (field data)** beetle catch: predicted effect sizes with trap loss at Treatments and Trapping Intervals of high beetle activity. Low replicate site and trap variance.

Figure S4. **Mean (field data)** beetle catch: predicted effect sizes with trap loss at Treatments and Trapping Intervals of high beetle activity. Low replicate site and high trap variance.

Figure S5. **Mean (field data)** beetle catch: predicted effect sizes with trap loss at Treatments and Trapping Intervals of high beetle activity. High replicate site and low trap variance.

Figure S6. **Mean (field data)** beetle catch: predicted effect sizes with trap loss at Treatments and Trapping Intervals of high beetle activity. High replicate site and trap variance.

-----"Low catch loss"-----

Figure S7. **Low mean** beetle catch: predicted effect sizes with trap loss at Treatments and Trapping Intervals of low beetle activity. Replicate site and trap variance from the field data.

Figure S8. **High mean** beetle catch: predicted effect sizes with trap loss at Treatments and Trapping Intervals of low beetle activity. Replicate site and trap variance from the field data.

Figure S9. **Mean (field data)** beetle catch: predicted effect sizes with trap loss at Treatments and Trapping Intervals of low beetle activity. Low replicate site and trap variance.

Figure S10. **Mean (field data)** beetle catch: predicted effect sizes with trap loss at Treatments and Trapping Intervals of low beetle activity. Low replicate site and high trap variance.

Figure S11. **Mean (field data)** beetle catch: predicted effect sizes with trap loss at Treatments and Trapping Intervals of low beetle activity. High replicate site and low trap variance.

Figure S12. **Mean (field data)** beetle catch: predicted effect sizes with trap loss at Treatments and Trapping Intervals of low beetle activity. High replicate site and trap variance.

-----"Random loss"-----

Figure S13. **Low mean** beetle catch: predicted effect sizes with random trap loss. Replicate site and trap variance from the field data.

Figure S14. **High mean** beetle catch: predicted effect sizes with random trap loss. Replicate site and trap variance from the field data.

Figure S15. **Mean (field data)** beetle catch: predicted effect sizes with random trap loss. Low replicate site and trap variance.

Figure S16. **Mean (field data)** beetle catch: predicted effect sizes with random trap loss. Low replicate site and high trap variance.

Figure S17. **Mean (field data)** beetle catch: predicted effect sizes with random trap loss. High replicate site and low trap variance.

Figure S18. **Mean (field data)** beetle catch: predicted effect sizes with random trap loss. High replicate site and trap variance.

Figure S1. High Catch Loss; Low Mean

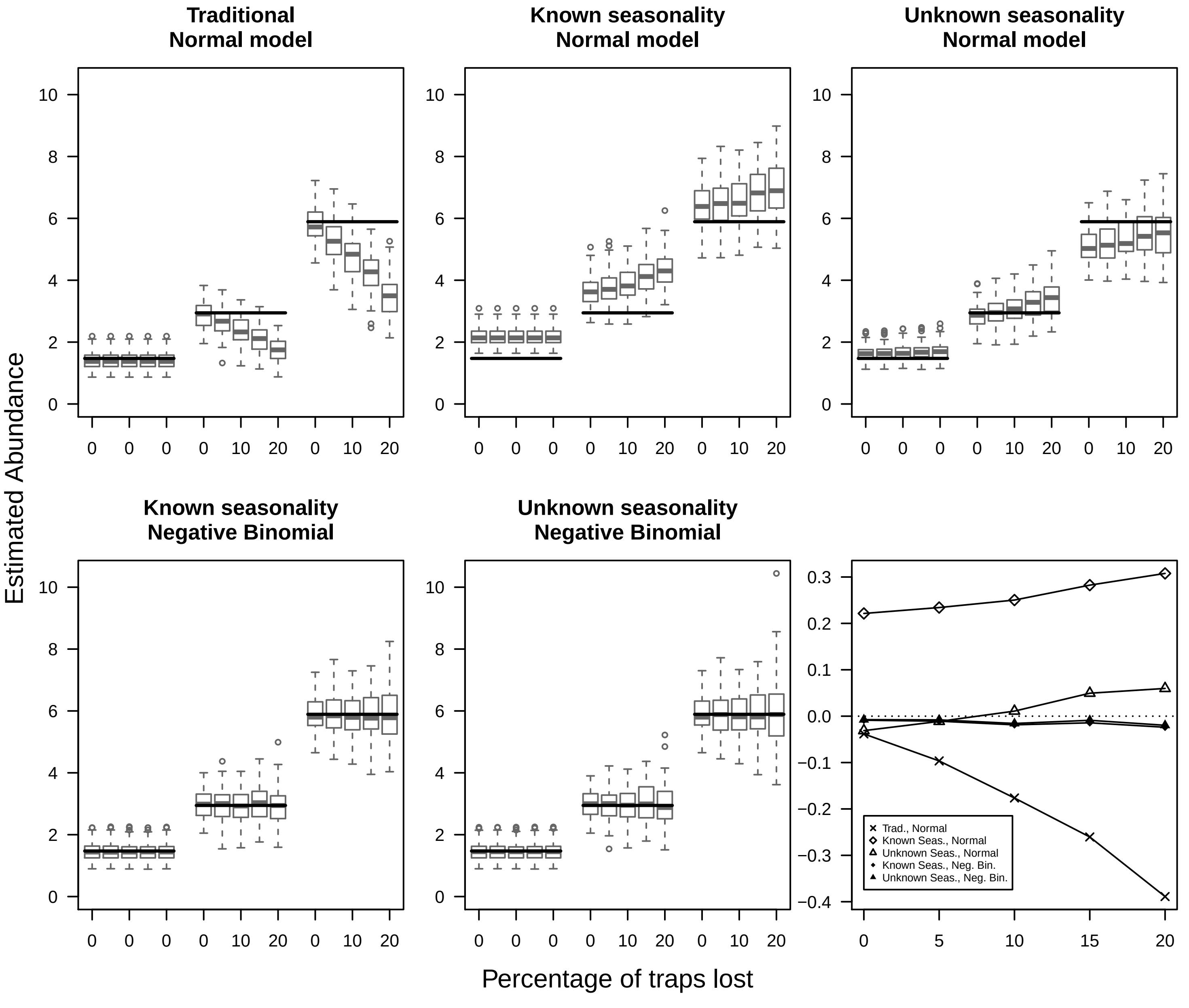

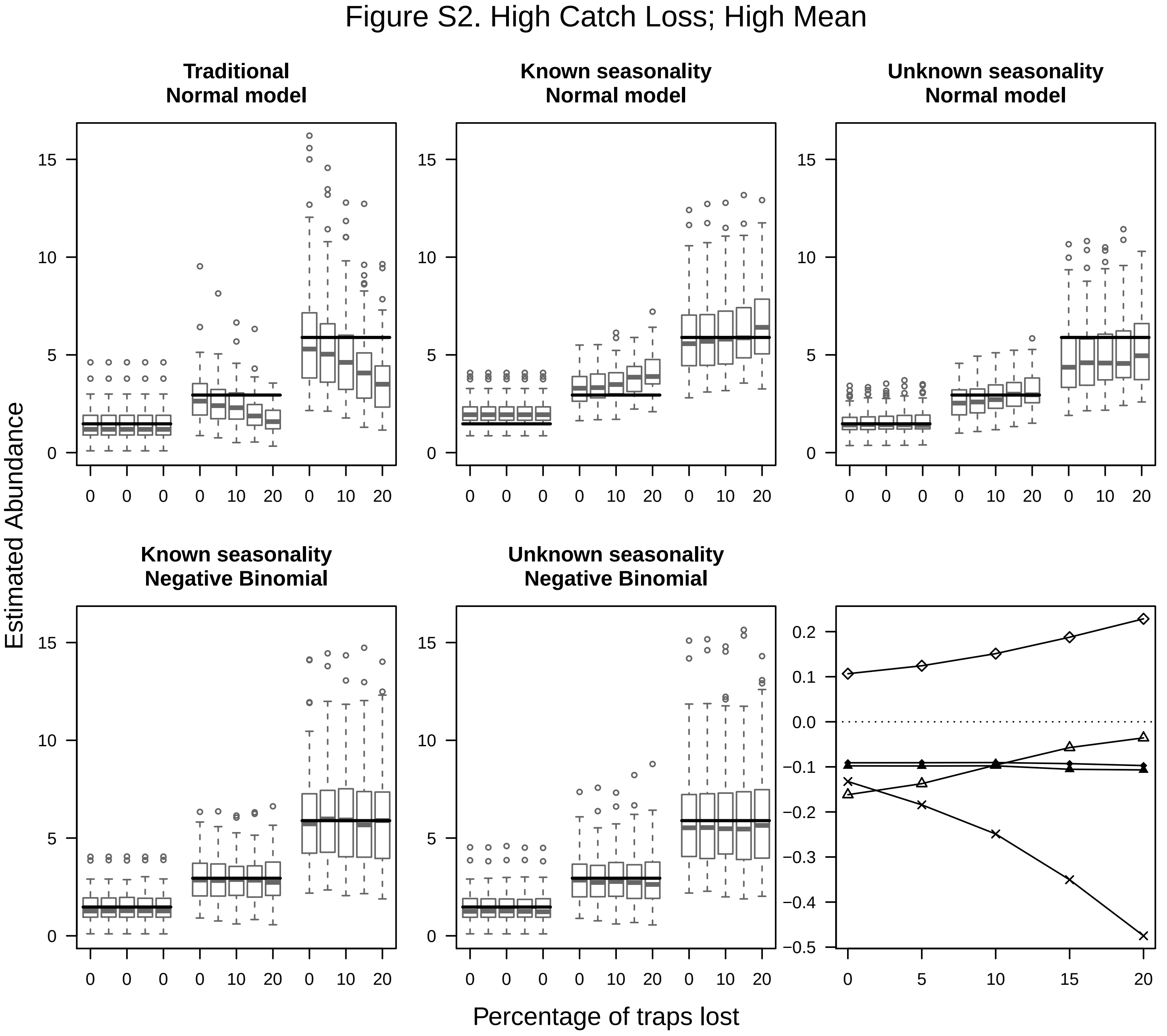
Figure S2. High Catch Loss; High Mean
Traditional
Normal model
Known seasonality
Normal model
Unknown seasonality
Normal model
Known seasonality
Negative Binomial
Unknown seasonality
Negative Binomial
Estimated Abundance
Percentage of traps lost

Figure S3. High Catch Loss; Low Site, Low Trap variance

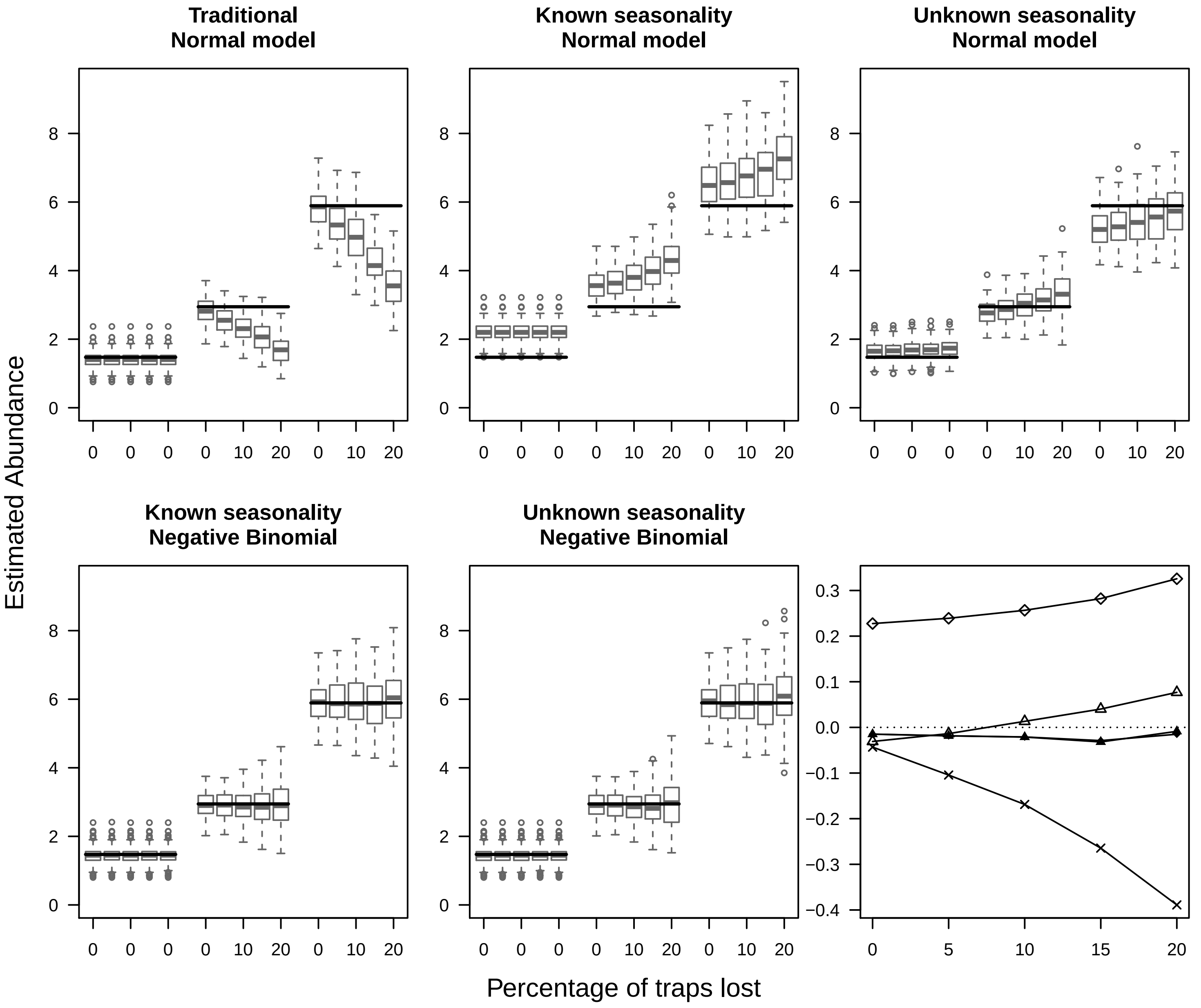

Figure S4. High Catch Loss; Low Site, High Trap variance

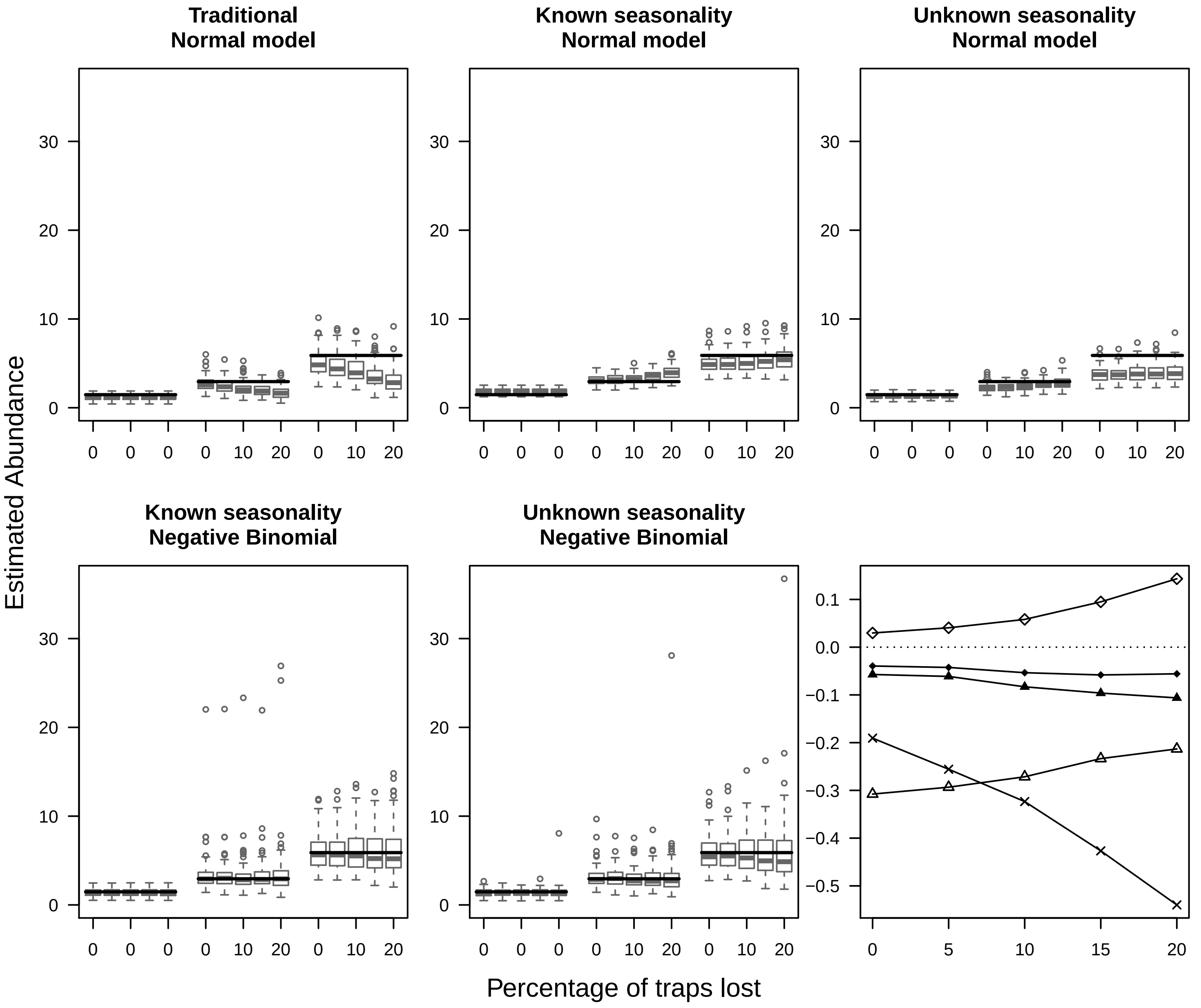

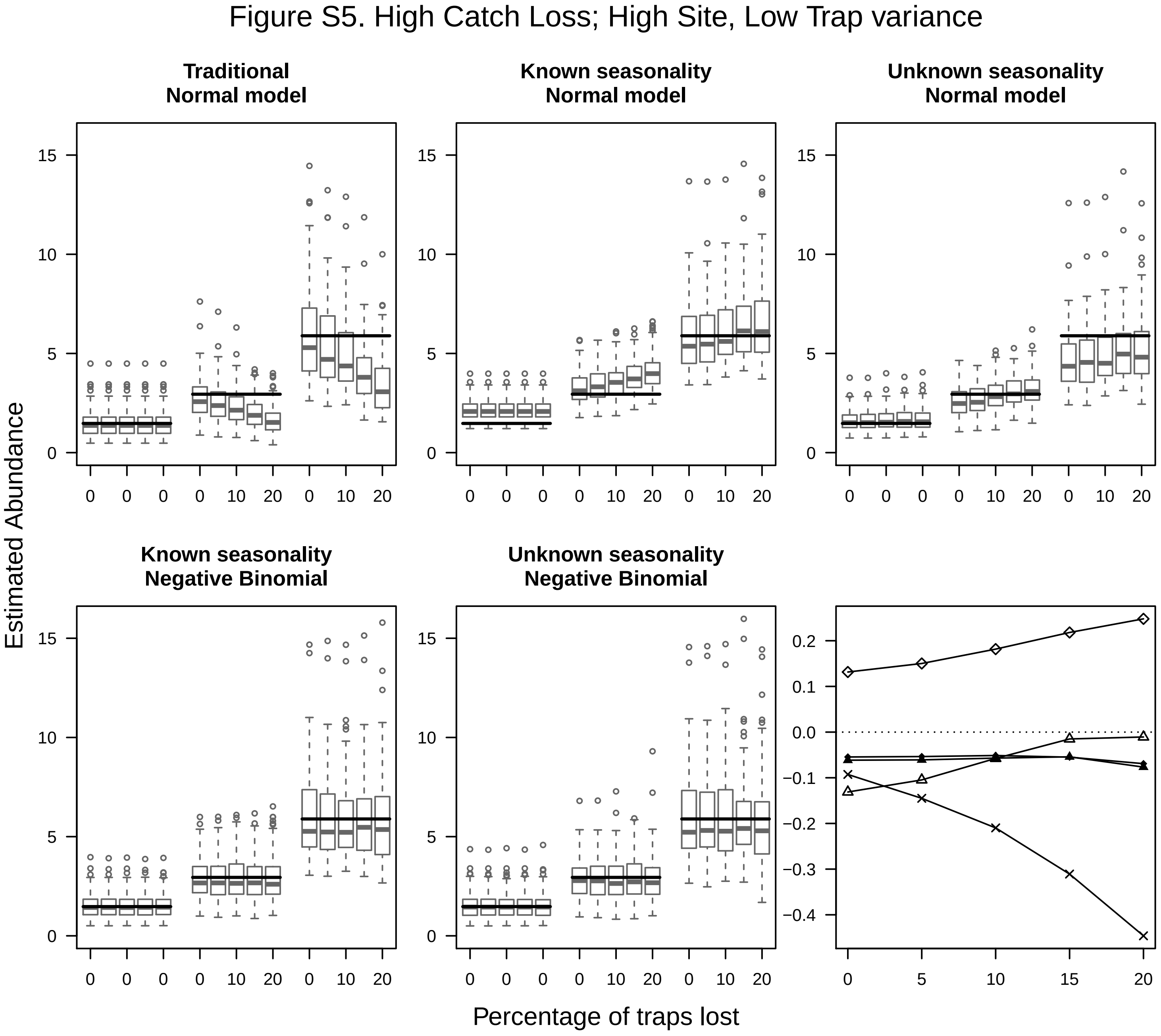

Figure S5. High Catch Loss; High Site, Low Trap variance
Traditional
Normal model
Known seasonality
Normal model
Unknown seasonality
Normal model
Known seasonality
Negative Binomial
Unknown seasonality
Negative Binomial
Estimated Abundance
Percentage of traps lost
0
5
10
15
20
0.2
0.1
0.0
-0.1
-0.2
-0.3
-0.4

Figure S6. High Catch Loss; High Site, High Trap variance

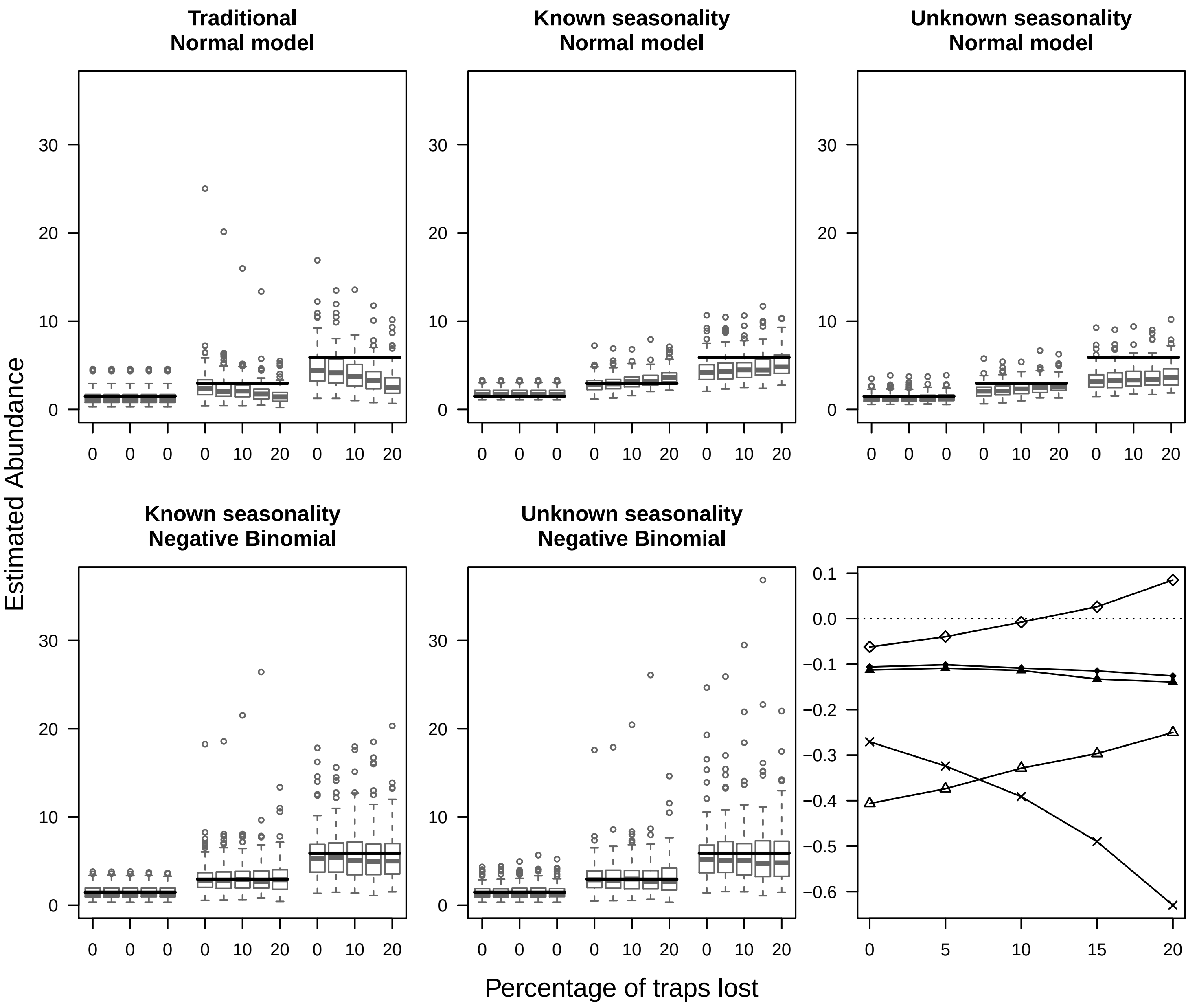

Figure S7. Low Catch Loss; Low Mean

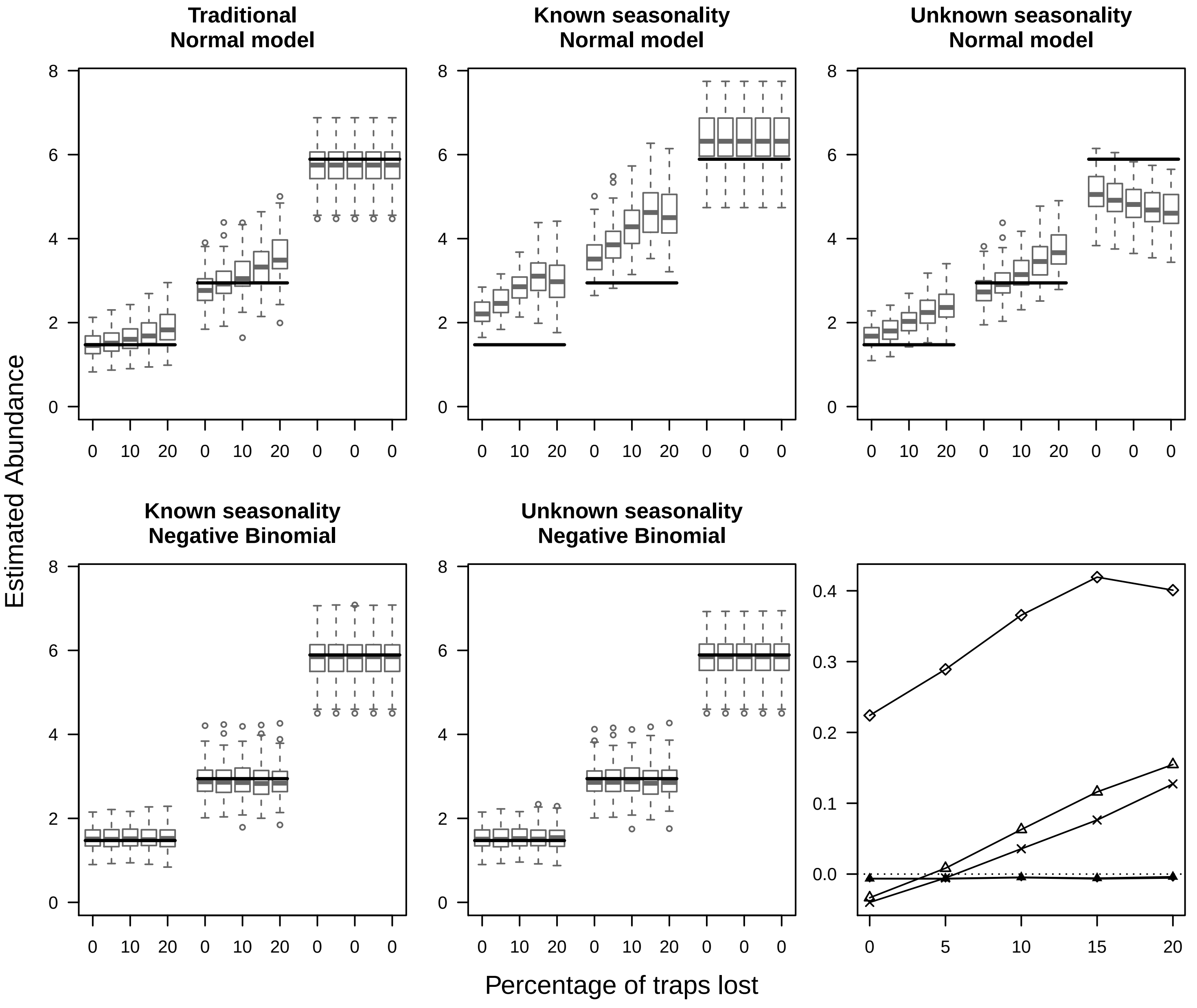

Figure S8. Low Catch Loss; High Mean

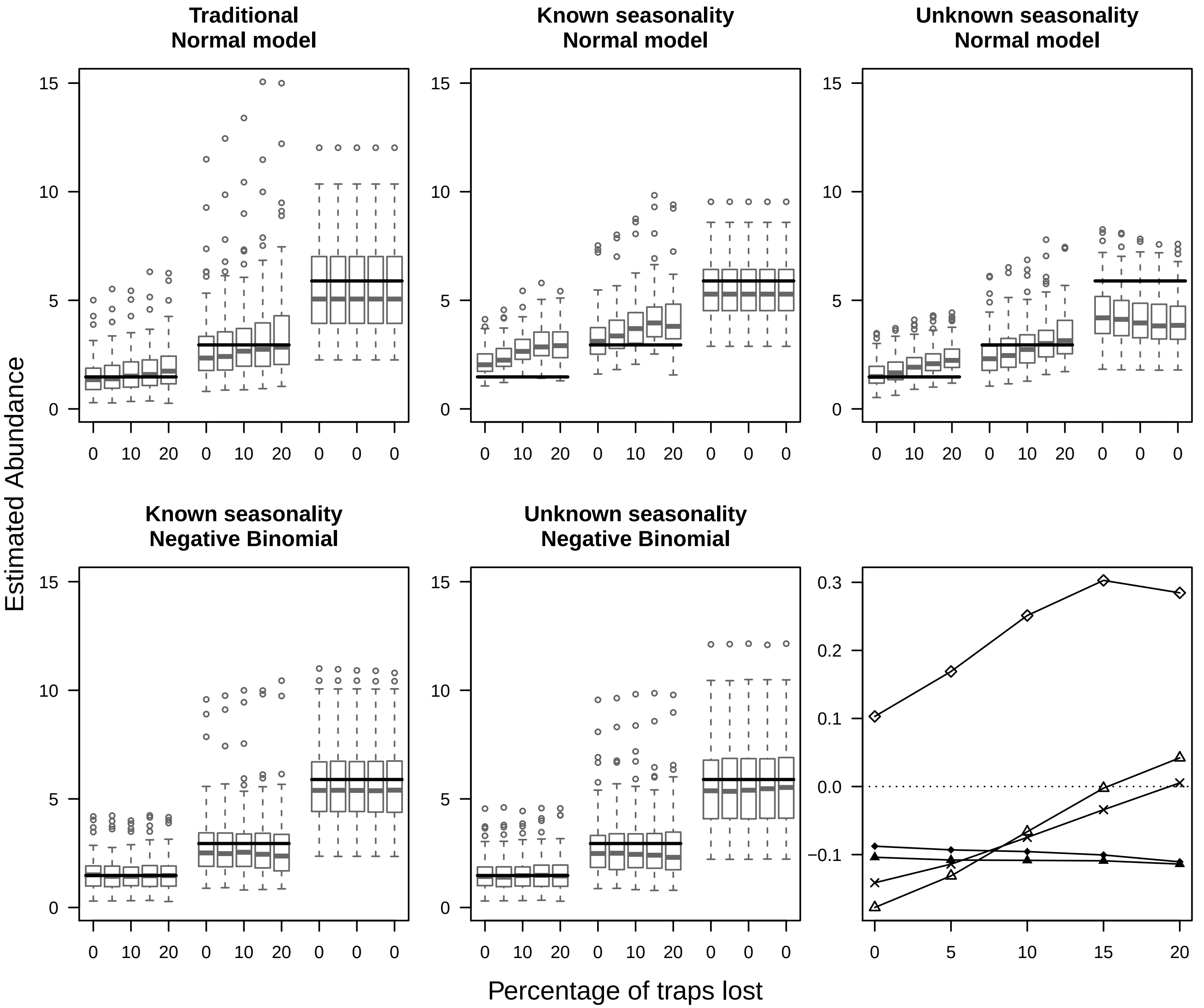

Figure S9. Low Catch Loss; Low Site, Low Trap variance

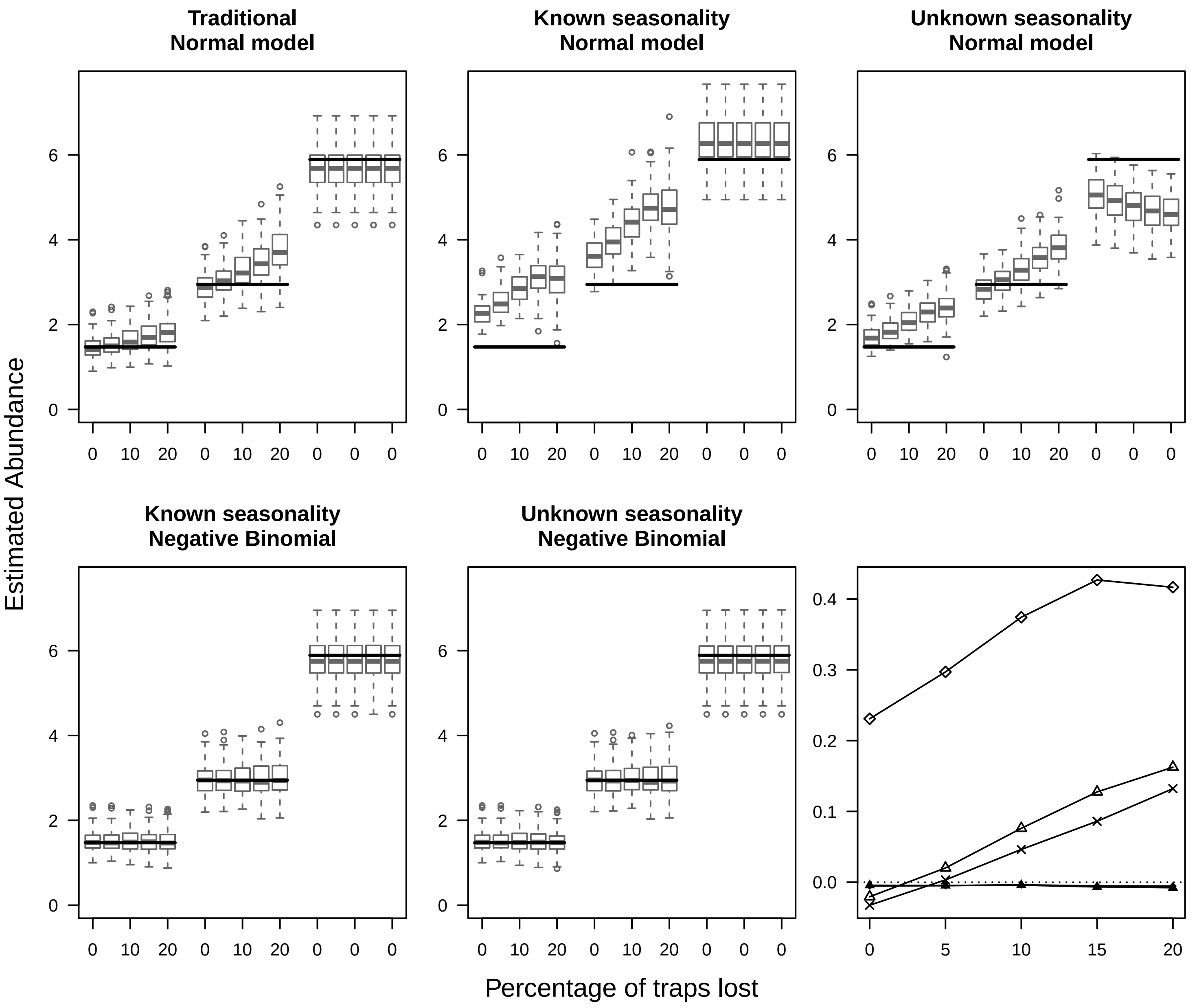

Figure S10. Low Catch Loss; Low Site, High Trap variance

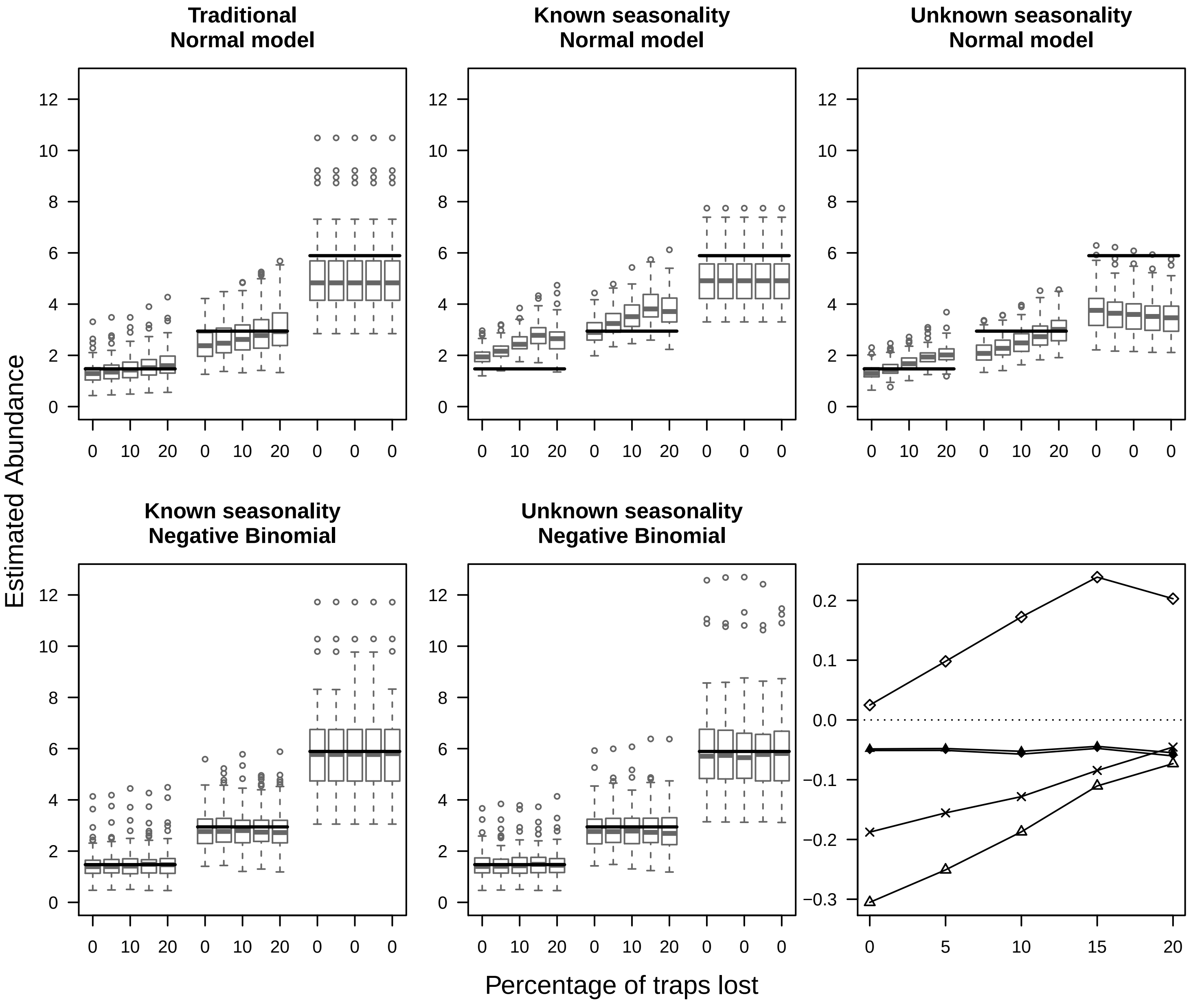

Figure S11. Low Catch Loss; High Site, Low Trap variance

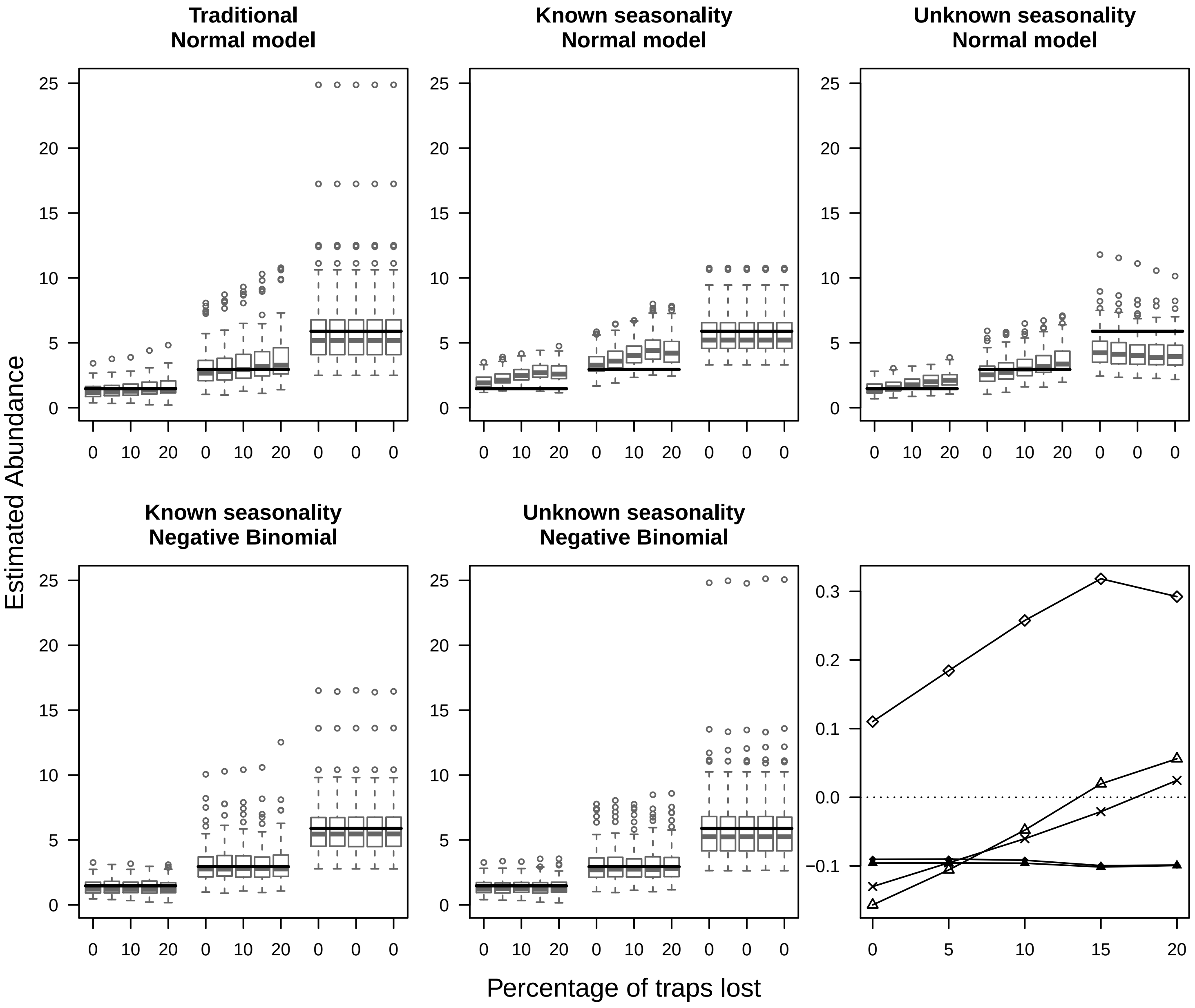

Figure S12. Low Catch Loss; High Site, High Trap variance

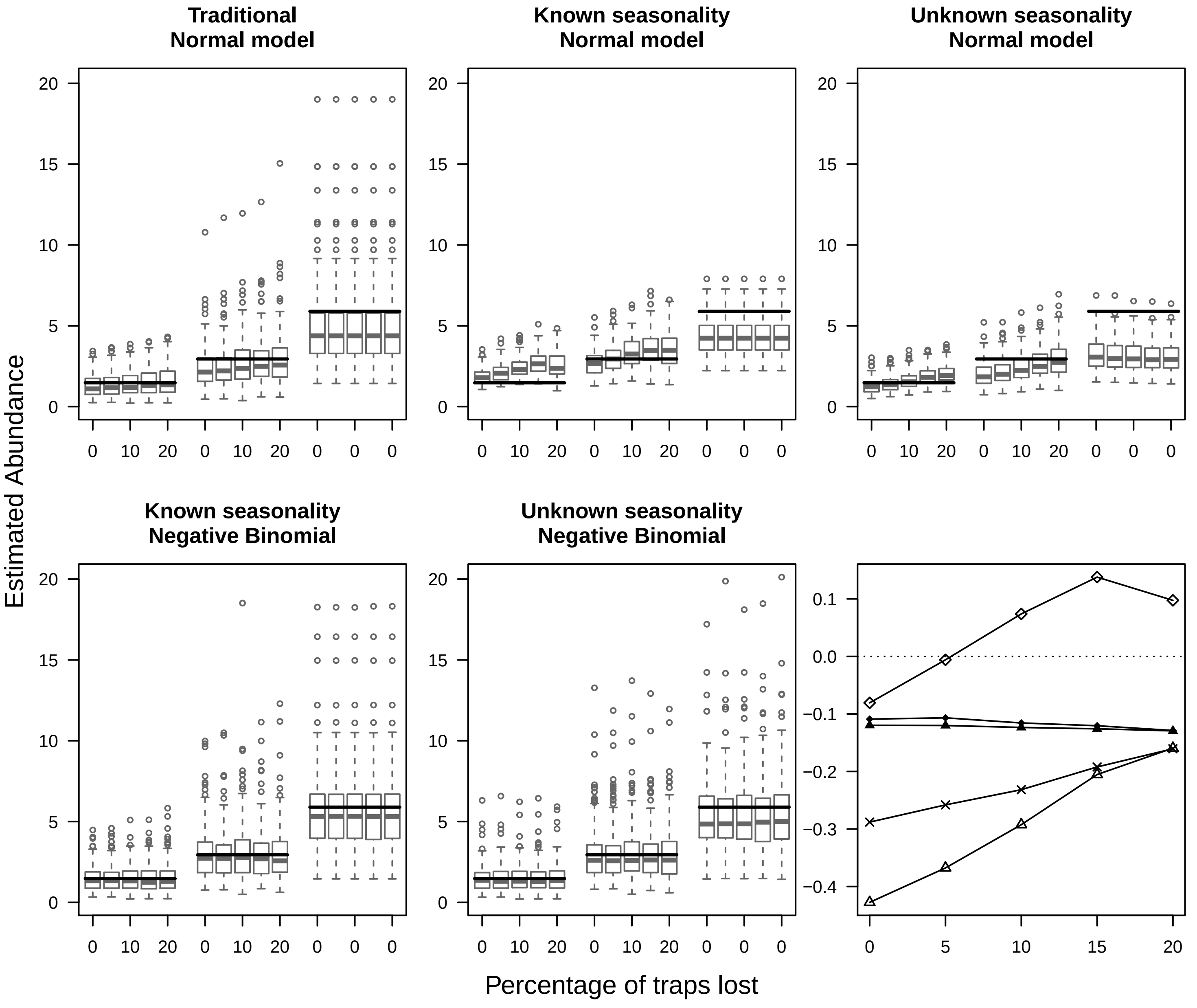

Figure S13. Random Loss; Low Mean

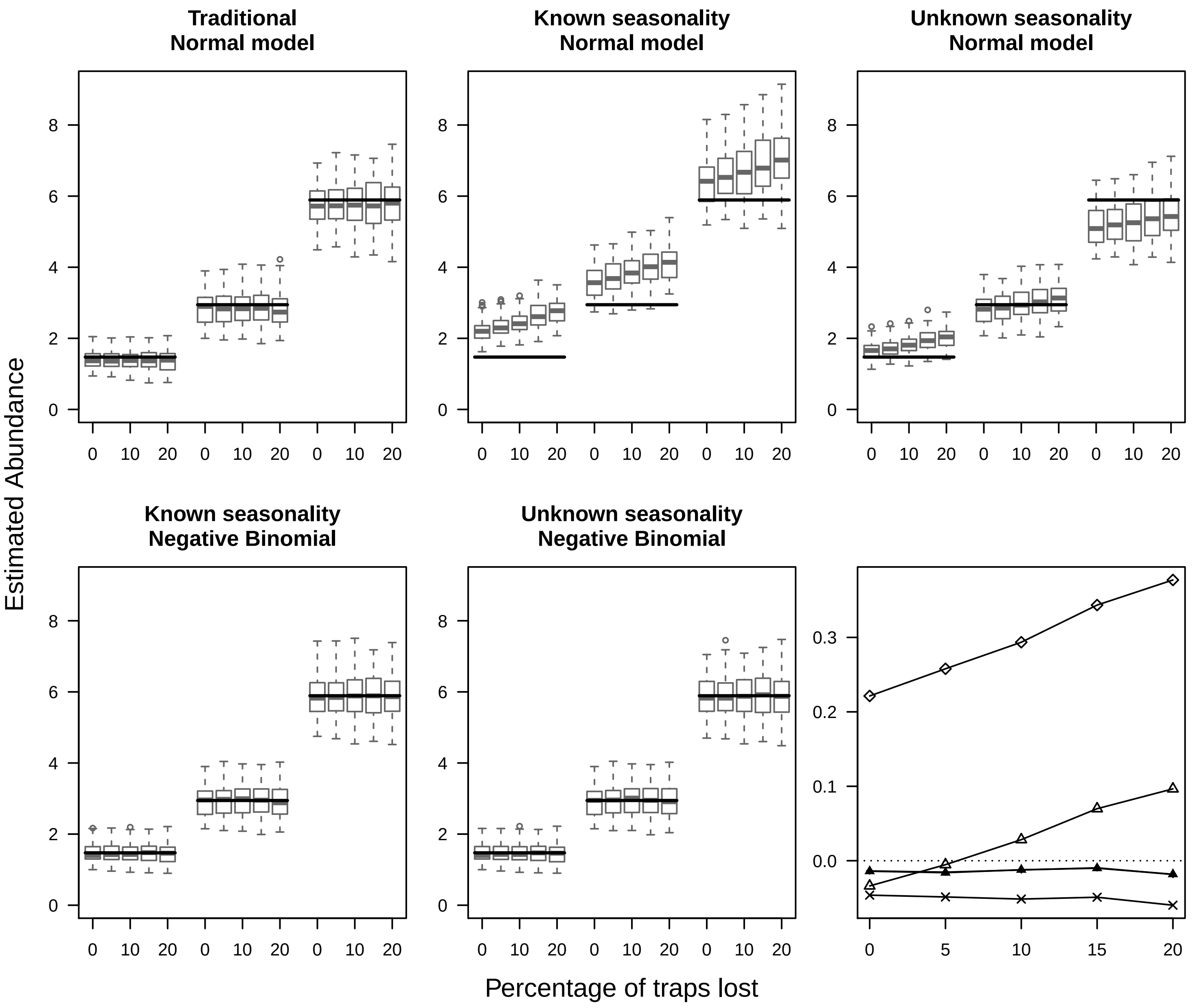

Figure S14. Random Loss; High Mean

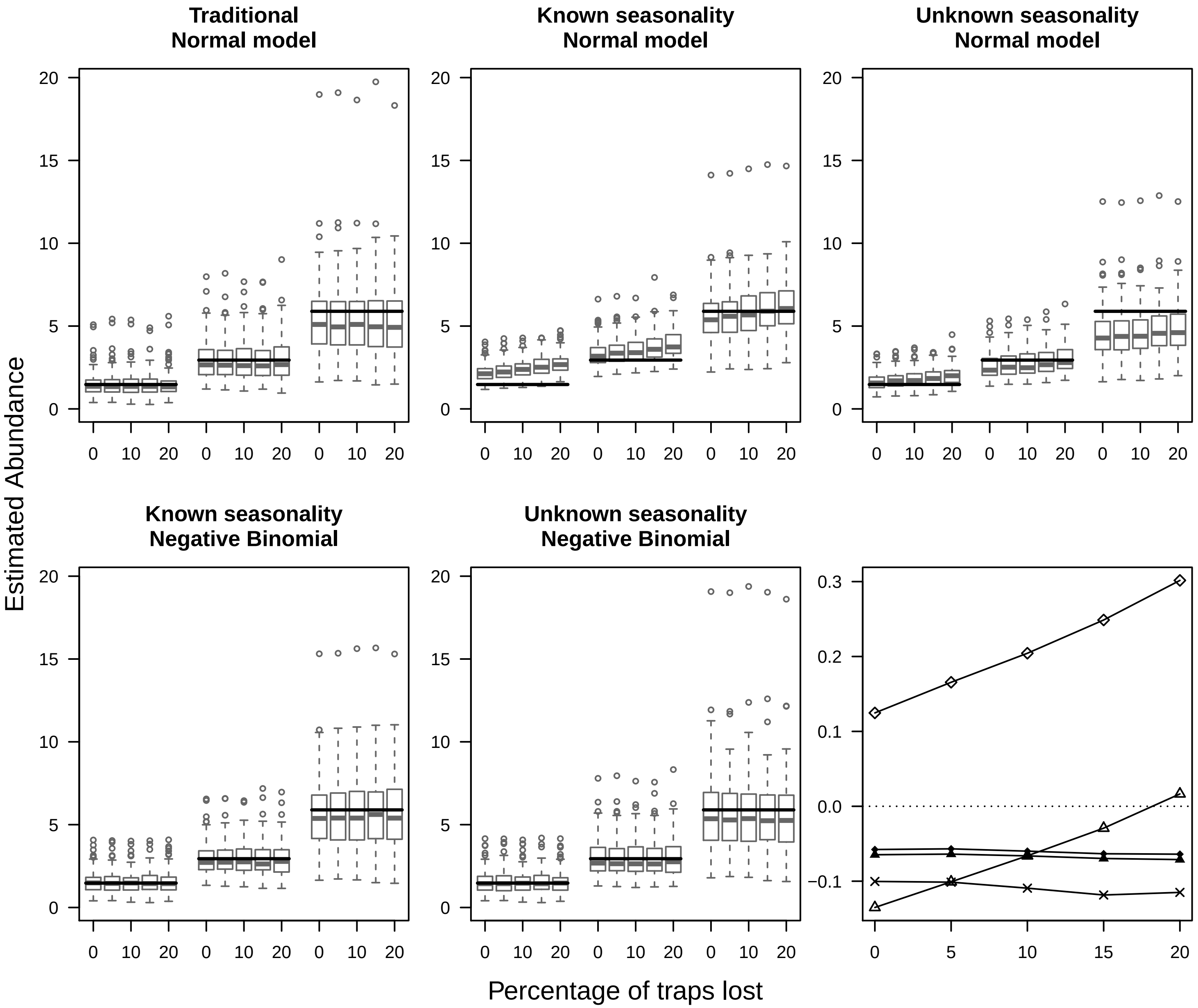

Figure S15. Random Loss; Low Site, Low Trap variance

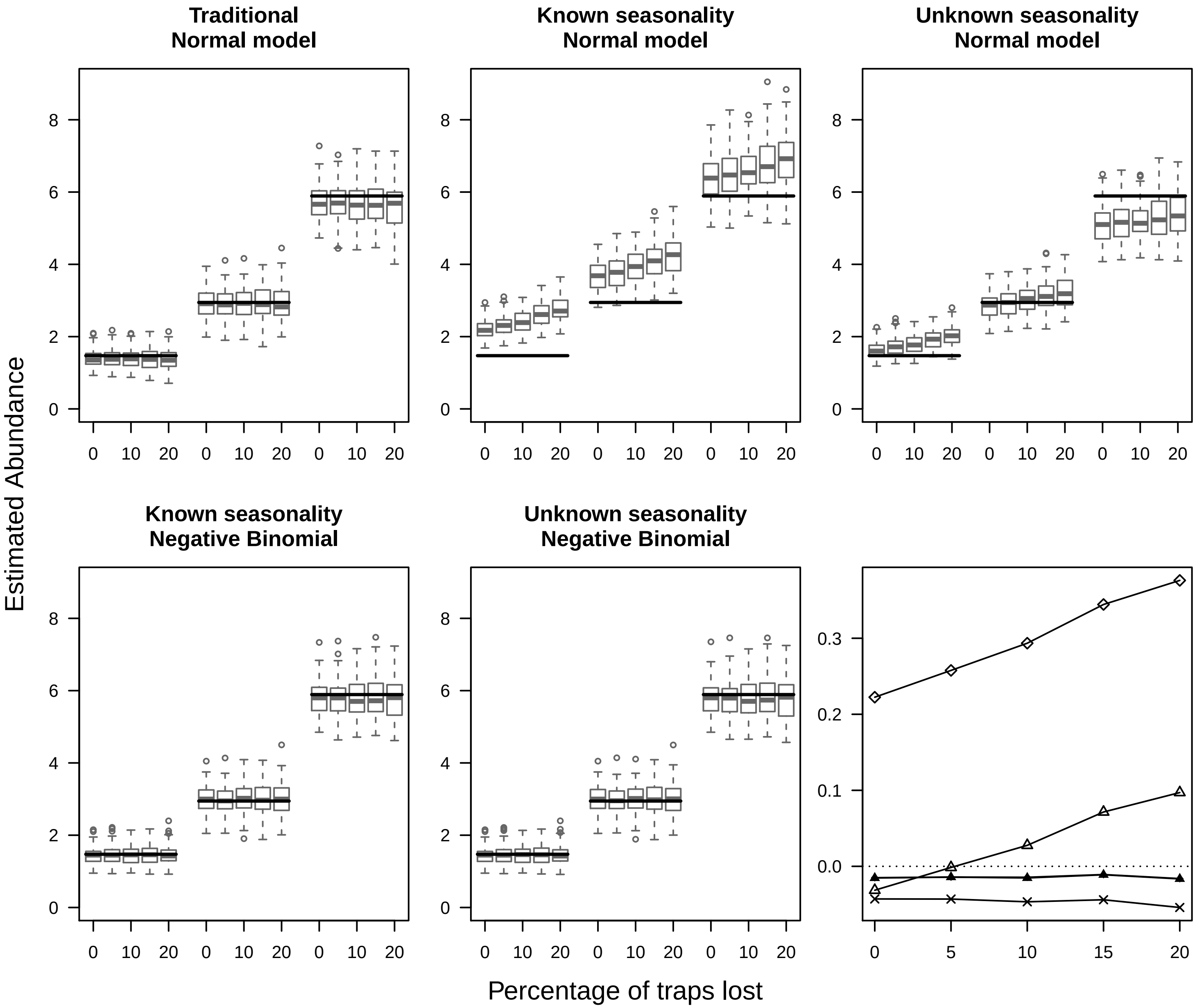

Figure S16. Random Loss; Low Site, High Trap variance

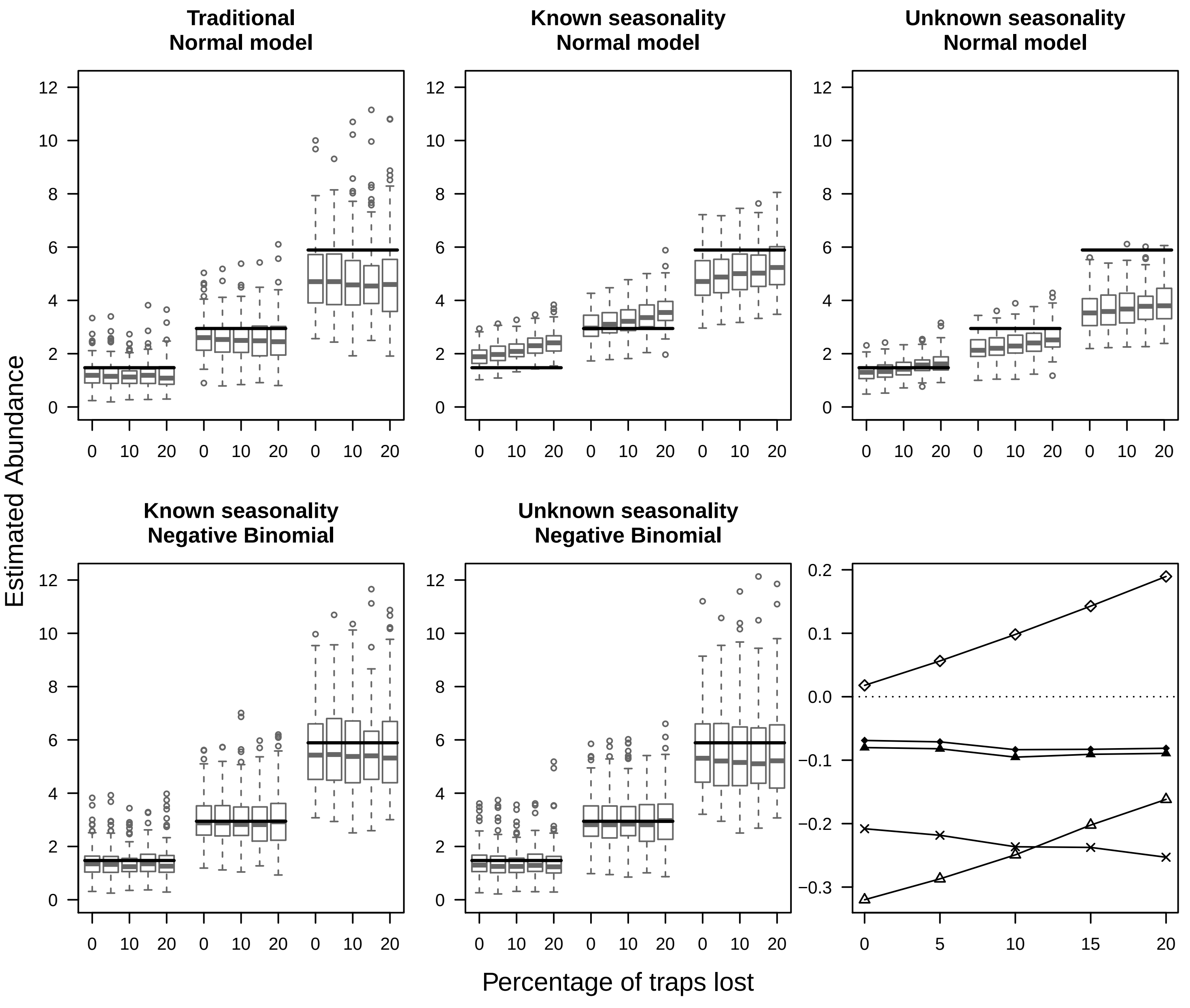

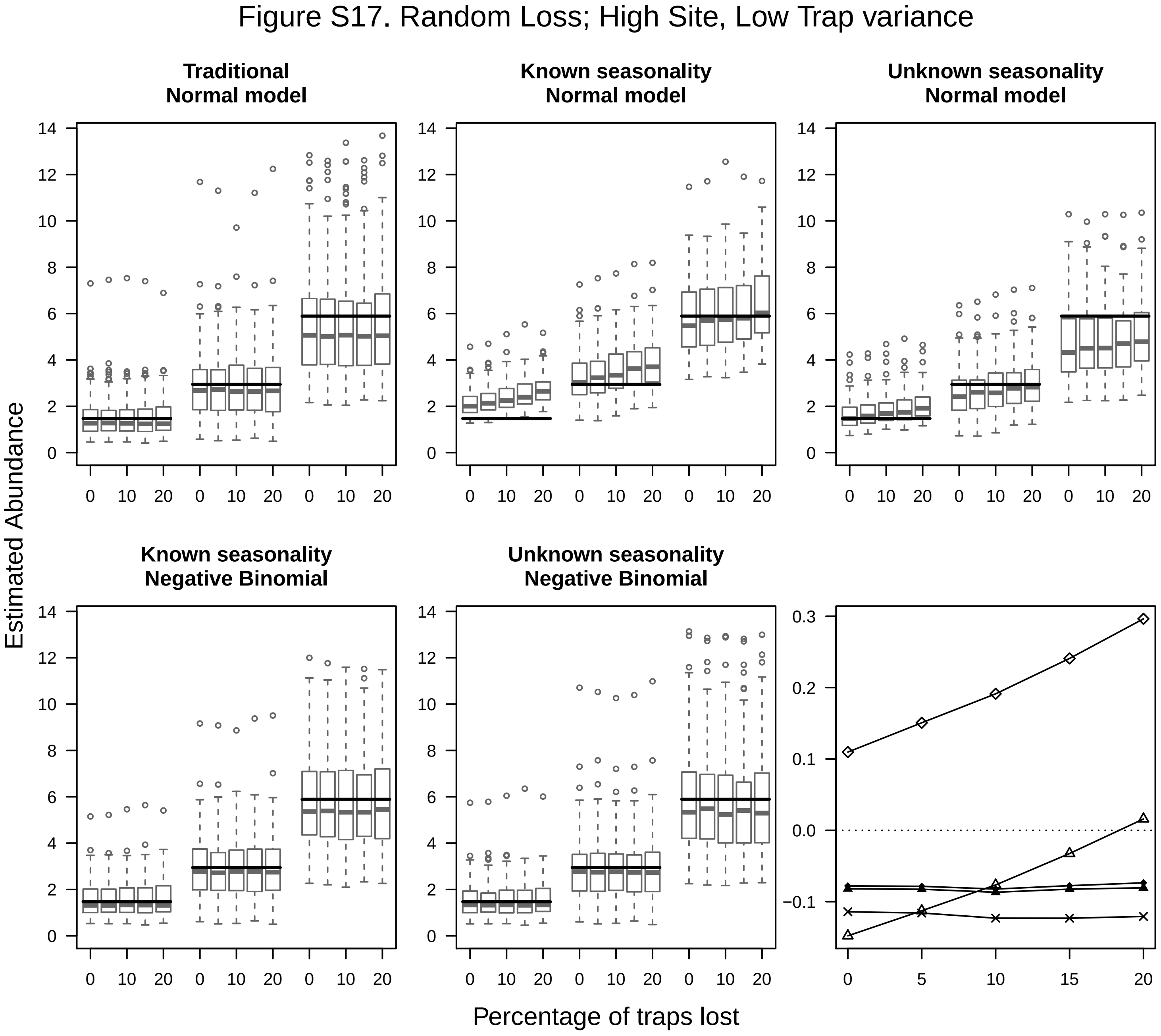
Figure S17. Random Loss; High Site, Low Trap variance
Traditional
Normal model
Known seasonality
Normal model
Unknown seasonality
Normal model
Known seasonality
Negative Binomial
Unknown seasonality
Negative Binomial
Estimated Abundance
Percentage of traps lost

Figure S18. Random Loss; High Site, High Trap variance

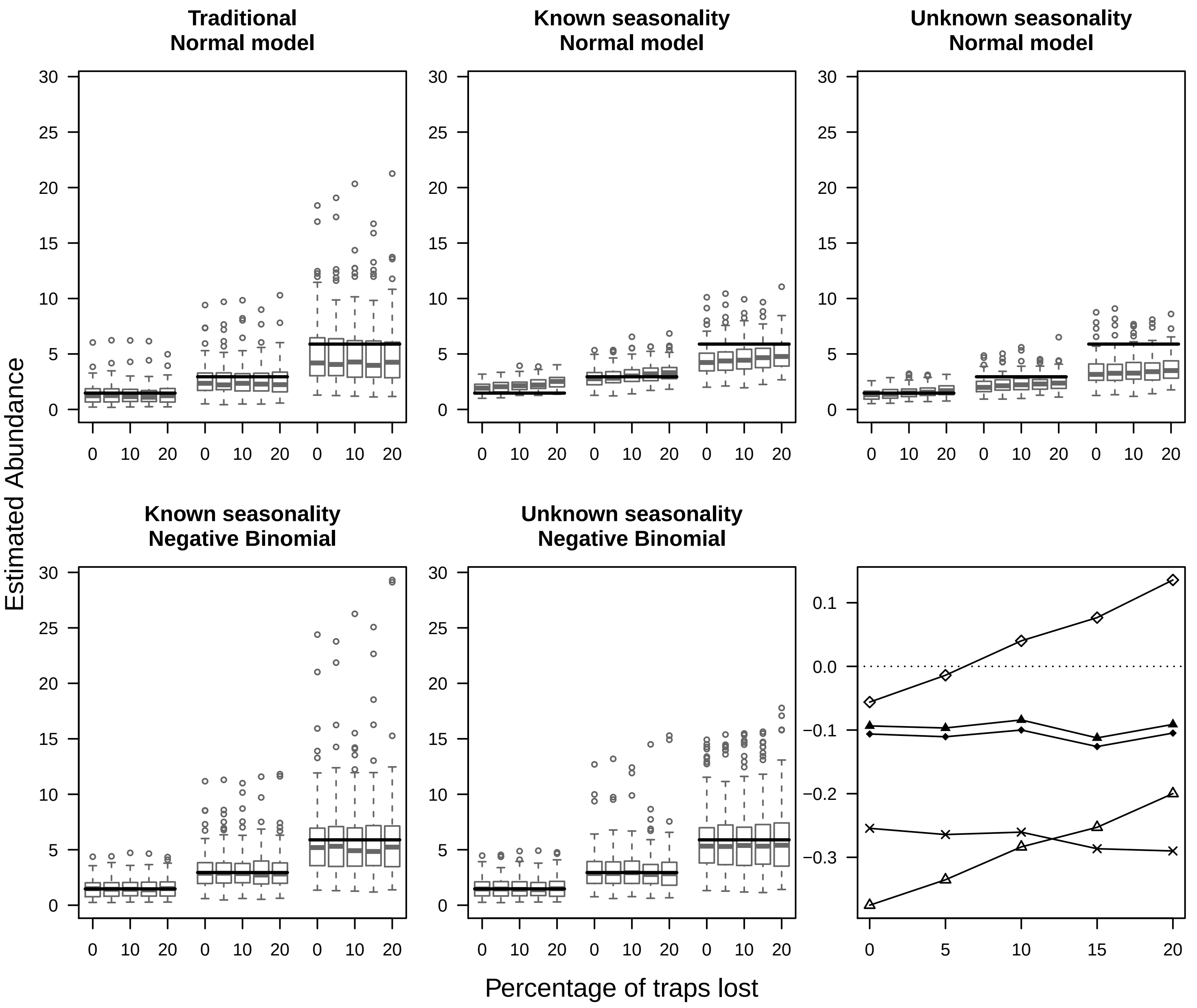

Supplement: Supporting Information S2 — Box and whisker plots of the effect sizes (predicted catch) of the analyses performed on the manipulated data (low and high means, low and high treatment site and trap variance, see Table 1 ). The black horizontal lines represent the simulated (i.e. true) total abundances per treatment without trap loss. The x-axis represents the three Treatment levels with five conditions per treatment (from no loss to 20% loss). Figs. S1–S6 are for trap losses at “High catch loss” (see Table 2). Figs. S7–S12 are for trap losses at “Low catch loss” (see Table 2). Figs. S13–S18 are for “Random trap losses”. The last panel in each figure represents the mean bias of the models against trap loss (see Fig. 3 in the manuscript for explanations). (PDF) [file pone.0040923.s002.pdf]
